# Geometry-informed irreversible perturbations for accelerated convergence of Langevin dynamics

**DOI:** 10.1007/s11222-022-10147-6

**Published:** 2022-09-19

**Authors:** Benjamin J. Zhang, Youssef M. Marzouk, Konstantinos Spiliopoulos

**Affiliations:** 1 Department of Aeronautics and Astronautics, Center for Computational Science and Engineering, Massachusetts Institute of Technology, Cambridge, USA; 2 Department of Mathematics and Statistics, Boston University, Boston, USA

**Keywords:** Monte Carlo sampling, Stochastic gradient Langevin dynamics, Riemannian manifold Langevin dynamics, Geometry-informed irreversibility, Bayesian computation

## Abstract

We introduce a novel geometry-informed irreversible perturbation that accelerates convergence of the Langevin algorithm for Bayesian computation. It is well documented that there exist perturbations to the Langevin dynamics that preserve its invariant measure while accelerating its convergence. Irreversible perturbations and reversible perturbations (such as Riemannian manifold Langevin dynamics (RMLD)) have separately been shown to improve the performance of Langevin samplers. We consider these two perturbations simultaneously by presenting a novel form of irreversible perturbation for RMLD that is informed by the underlying geometry. Through numerical examples, we show that this new irreversible perturbation can improve estimation performance over irreversible perturbations that do not take the geometry into account. Moreover we demonstrate that irreversible perturbations generally can be implemented in conjunction with the stochastic gradient version of the Langevin algorithm. Lastly, while continuous-time irreversible perturbations cannot impair the performance of a Langevin estimator, the situation can sometimes be more complicated when discretization is considered. To this end, we describe a discrete-time example in which irreversibility increases both the bias and variance of the resulting estimator.

## Introduction

Bayesian inference often requires estimating expectations with respect to non-Gaussian distributions. To solve this problem, particularly in high dimensions, one frequently resorts to sampling methods. A commonly used class of sampling methods is based on the Langevin dynamics (LD), which uses the gradient of the log-target density to specify a stochastic differential equation (SDE) whose invariant distribution is the target (e.g., posterior) distribution of interest. Long term averages over a single trajectory of the SDE can be then used to estimate expectations of interest by appealing to the ergodicity of the stochastic process. Other LD-based approaches that reduce the mean squared error (MSE) of such estimators include the Metropolis-adjusted Langevin algorithm (MALA) (Roberts and Tweedie 1996; Girolami and Calderhead 2011), the stochastic gradient Langevin dynamics (SGLD) (Welling and Teh 2011), and their variants.

It is also known that certain perturbations to the LD can accelerate convergence of the dynamics to the stationary distribution. In Rey-Bellet and Spiliopoulos (2015) the authors show that suitable reversible and irreversible perturbations to diffusion processes can decrease the spectral gap of the generator, as well as increase the large deviations rate function and decrease the asymptotic variance of the estimators. One widely celebrated choice of *reversible* perturbation is the Riemannian manifold Langevin dynamics (Girolami and Calderhead 2011), in which one defines a Riemannian metric to alter the way distances and gradients are computed. The use of *irreversible* perturbations to accelerate convergence has also been well studied in a variety of contexts and general settings (Rey-Bellet and Spiliopoulos 2015; Hwang et al. 2005; Rey-Bellet and Spiliopoulos 2015, 2016); see also (Franke et al. 2010; Hwang et al. 1993; Bierkens 2016; Diaconis et al. 2010) and for linear systems, Lelievre et al. (2013). The authors of Ma et al. (2015) find general conditions on the drift and diffusion coefficients of an SDE so that a specified measure is the SDE’s invariant measure—without, however, exploring how different choices of these coefficients impact sampling quality. Existing literature shows that augmenting the drift of the LD with a vector field that is orthogonal to the gradient of the log-target density will leave the invariant measure unchanged while decreasing the spectral gap. A convenient choice is simply to add the vector field induced by a skew-symmetric matrix applied to the gradient of the log posterior.

At the same time, traditional sampling methods for Bayesian inference are often intractable for extremely large datasets. While Langevin dynamics-based sampling methods only require access to the unnormalized posterior density, they need many evaluations of this unnormalized density and its gradient. When the dataset is extremely large, each evaluation of the density may be computationally intractable, as it requires the evaluation of the likelihood over the entire dataset. In the past decade the *stochastic gradient* Langevin dynamics (SGLD) has been introduced and analyzed (Welling and Teh 2011; Teh et al. 2016) to address the problem posed by large datasets. Rather than evaluating the likelihood over the entire dataset, SGLD subsamples a portion of the data (either with or without replacement) and uses the likelihood evaluated at the sampled data to estimate the true likelihood. The resulting chain can then be used to estimate ergodic averages.

In this paper we present a *state-dependent irreversible* perturbation of Riemannian manifold Langevin dynamics that is informed by the *geometry* of the manifold. This departs from existing literature, as the vector field of the resulting perturbation is *not* orthogonal to the original drift term. This geometry-informed irreversible perturbation accelerates convergence and, if desired, can be used in combination with the SGLD algorithm to exploit the computational savings of a stochastic gradient.

We demonstrate this approach on a variety of examples: a simple anisotropic Gaussian target, a posterior on the mean and variance parameters of a normal distribution, Bayesian logistic regression, and Bayesian independent component analysis (ICA). Generally, we observe that the geometry-informed irreversible perturbation improves the convergence rate of LD compared to a standard irreversible perturbation. The improvement tends to be more pronounced as the target distribution deviates from Gaussianity. Our numerical studies also show that introducing irreversibility can reduce the MSE of the resulting long-term average estimator, mainly by reducing variance. In many cases this reduction can be significant, e.g., 1–2 orders of magnitude.

One must, however, also take the effects of discretization into account. In the continuous-time setting, it is known theoretically that irreversible perturbations can at worst only leave the spectral gap fixed. In borderline cases, though—i.e., in cases where the continuous-time theoretical improvement is nearly zero—after accounting for discretization, stiffness can actually cause the resulting estimator to perform worse than if no irreversibility were applied at all. Indeed, we will describe in Appendix A an illustrative Gaussian example in which the standard Langevin algorithm performs better than the algorithm with the standard irreversible perturbation—that is, an example in which additional irreversibility leads to increased bias and variance of the long term average estimator (see Remark 3.2 and Remark A.1 for a theoretical explanation). Along similar lines, the idea of applying irreversible perturbations to SGLD has recently been studied in the context of nonconvex stochastic optimization (Hu et al. 2020). The authors also note that while irreversibility increases the rate of convergence, it increases the discretization error and amplifies the variance of the gradient, compared to a non-perturbed system with the same step size; see also (Brosse et al. 2018) for a related discussion on the relation of SGLD to SGD and convergence properties. This reflects the increased stiffness of irreversible SGLD relative to standard SGLD.

The rest of the paper is organized as follows. In Section 2 we review reversible and irreversible perturbations of the overdamped Langevin dynamics that may improve the efficiency of sampling from equilibrium. Then, in Section 2.3, we present our new geometry-informed irreversible perturbation. In Section 3 we present simulation studies that demonstrate the good performance of this geometric perturbation, relative to a variety of other standard reversible and irreversible choices. In several of these examples, we also demonstrate the use of stochastic gradients. Section 4 summarizes our results and outlines directions for future work. Appendix A details the simple Gaussian example showing that in “borderline” cases—i.e., when continuous-time analysis does not predict improvements from irreversible perturbations—the stiffness created by an irreversible perturbation can, after discretization, lead to poorer performance than the unperturbed case.

## Improving the performance of Langevin samplers

We begin by recalling some relevant background on Langevin samplers, Riemannian manifold Langevin dynamics, perturbations of Langevin dynamics, and the stochastic gradient Langevin dynamics algorithm. Let $f(\theta )$ be a test function on state space $E \subset {\mathbb {R}}^d$ and let $\pi (\theta )$ be some unnormalized target density on *E*. In our experiments, $\pi (\theta )$ arises as a posterior density of the form $\pi (\theta ) \propto L(\theta ;X) \pi _0(\theta )$, where $L(\theta ; X)$ is the likelihood model, *X* are the data, and $\pi _0(\theta )$ is the prior density. Define $\{\theta (t)\}$ as a Langevin process that has invariant density $\pi (\theta )$:

1 $$\begin{aligned} \text {d}\theta (t) = \beta \nabla \log \pi (\theta (t)) \text {d}t + \sqrt{2\beta }\text {d}W(t), \end{aligned}$$

where $\beta >0$ denotes the temperature, *W*(*t*) is a standard Brownian motion in ${\mathbb {R}}^d$, and the initial condition may be arbitrary. By ergodicity, we may compute expectations with respect to the posterior by the long term average of $f(\theta )$ over a single trajectory:

2 $$\begin{aligned} {\mathbb {E}}_\pi [f(\theta )] = \int _E f(\theta ) \pi (\theta ) \text {d}\theta = \lim _{t\rightarrow \infty } \frac{1}{T} \int _0^T f(\theta (t)) \text {d}t. \end{aligned}$$

For practical computations, we must approximate (2) by discretizing the Langevin dynamics and choosing a large but finite *T*. Applying the Euler-Maruyama method to (1) with step size *h* yields the following recurrence relation,

3 $$\begin{aligned} \theta _{k+1} = \theta _k + h\beta \nabla \log \pi (\theta _{k}) \text {d}t + \sqrt{2\beta h} \xi _{k+1} \end{aligned}$$

where $\xi _{k}$ are independent standard normal random variables. The total number of steps is equal to $K = T/h$. The resulting estimator for (2) is

4 $$\begin{aligned} {\mathbb {E}}_\pi [f(\theta )] \approx \frac{1}{K} \sum _{k = 0}^{K-1} f(\theta _k). \end{aligned}$$

This estimator is the *unadjusted Langevin algorithm* (ULA), which has found renewed interest in the context of high-dimensional machine learning problems (Durmus and Moulines 2019). Discretization and truncation, however, introduce bias into the estimator. Moreover, there are noted examples in which the continuous-time process and the discretized version do not have the same invariant distribution no matter the choice of the fixed, but nonzero, discretization step *h*; see Ganguly and Sundar (2021) for a related discussion. Certain Markov chain Monte Carlo (MCMC) methods such as MALA circumvent these issues by using the dynamics to propose new points, but accepting or rejecting them according to some rule so that the resulting discrete-time Markov chain has the target distribution as its invariant distribution (Roberts and Tweedie 1996; Girolami and Calderhead 2011).

Many different SDEs can have the same invariant distribution. Therefore, there has been much study into how the standard Langevin dynamics of some target distribution can be altered to increase its rate of convergence. Some examples of this can be found in the work of Hwang et al. (2005), Rey-Bellet and Spiliopoulos (2016) and others. The standard Langevin dynamics is a reversible Markov process, meaning that the process satisfies detailed balance. The work of Rey-Bellet and Spiliopoulos (2016) studies, in general terms, how reversible and irreversible perturbations to reversible processes decrease the spectral gap, increase the large deviations rate function, and decrease the asymptotic variance. Yet how to *choose* such perturbations to most efficiently accelerate convergence is yet to be thoroughly studied in settings beyond linear diffusion processes (Lelievre et al. 2013). Also, with the exception of a few examples—see for instance (Duncun et al. 2017; Jianfeng and Spiliopoulos 2018)—these perturbations have mainly been studied in the continuous-time setting.

### Reversible perturbations and Riemannian manifold Langevin dynamics

We only review relevant aspects of reversible perturbations and RMLD in this section. For a detailed review of RMLD and its related Monte Carlo methods, we refer the reader to Girolami and Calderhead (2011), Livingstone and Girolami (2014), Xifara et al. (2014). Let ${\textbf {B}}(\theta )$ be a $d\times d$ symmetric positive definite matrix. A reversible perturbation on LD (1) is an SDE with multiplicative noise:

5 $$\begin{aligned} \text {d}\theta (t)&= \beta \left[ \mathbf {B}(\theta )\nabla \log \pi (\theta (t)) +\nabla \cdot \mathbf {B}(\theta ) \right] \, \text {d}t \nonumber \\&\quad + \sqrt{2\beta \mathbf {B}(\theta )}\text {d}W(t). \end{aligned}$$

Here, the *i*-th component of $\nabla \cdot \mathbf {B}(\theta )$ is $\sum _{j = 1}^d \partial _{\theta _{j}} \mathbf {B}_{ij}(\theta )$. This is equivalent to Langevin dynamics defined on a Riemannian manifold, where the metric is $\mathbf {G}(\theta ) = \mathbf {B}(\theta )^{-1}$ (Xifara et al. 2014). A straightforward calculation shows that (5) with $\mathbf {B}(\theta )$ being any symmetric positive-definite matrix admits the same invariant distribution, $\pi$. The improved rate of convergence depends on the choice of the underlying metric. The work of Girolami and Calderhead (2011) argues that choosing the expected Fisher information matrix plus the Hessian of the log-prior to be the metric improves the performance of the resulting manifold MALA method. Meanwhile, Rey-Bellet and Spiliopoulos (2016) shows that under certain regularity conditions, if $\mathbf {B}(\theta )$ is chosen such that $\mathbf {B}(\theta ) - \mathbf {I}$ is positive definite, then the resulting estimator is expected to have improved performance in terms of the asymptotic variance, the spectral gap, and the large deviations rate function.

### Irreversible perturbations

Consider the following Langevin dynamics

6 $$\begin{aligned} \text {d}\theta (t) = \left[ \beta \nabla \log \pi (\theta (t)) +\gamma (\theta (t)) \right] \text {d}t +\sqrt{2\beta } \text {d}W(t). \end{aligned}$$

When $\gamma (\theta ) \equiv 0$, the process is reversible and has $\pi (\theta )$ as its invariant distribution. If $\gamma \ne 0$, then the resulting process will, in general, be time-irreversible unless $\gamma (\theta )$ can be written as a multiple of $\nabla \log \pi (\theta )$; see for example (Pavliotis 2014). However, an irreversible perturbation can still preserve the invariant distribution of the system. By considering the Fokker-Planck equation, one can show that if $\gamma (\theta )$ is chosen such that $\nabla \cdot \left( \gamma \pi \right) = 0$, then $\pi$ will still be the invariant distribution. A frequently used choice in the literature is $\gamma (\theta ) = \mathbf {J}\nabla \log \pi (\theta )$, where $\mathbf {J}$ is a constant skew-symmetric matrix, i.e., $\mathbf {J}= -\mathbf {J}^T$. The computational advantage of this choice is clear since only one additional matrix-vector multiply is needed to implement it. The optimal choice of irreversible perturbation to a linear system (i.e., that which yields fastest convergence) was completely analyzed in Lelievre et al. (2013).

The advantages of using irreversible perturbations is widely noted. The main result of Hwang et al. (2005) is that under certain conditions, the spectral gap, i.e., the difference between the leading two eigenvalues of the generator of the Markov semigroup, increases when $\gamma \ne 0$. In Rey-Bellet and Spiliopoulos (2015, 2015, 2016), the large deviations rate function is introduced as a measure of performance in the context of sampling from the equilibrium, and upon connecting it to the asymptotic variance of the long term average estimator, it is proven that adding an appropriate perturbation $\gamma$ not only increases the large deviations rate function but also decreases the asymptotic variance of the estimator. The use of irreversible proposals in the MALA was studied in Ottobre et al. (2019).

### Irreversible perturbations for RMLD

In this section, we will introduce our novel geometry-informed irreversible perturbation to Langevin dynamics. Suppose that we are given a diffusion process as in (5), and we want to study how to choose an irreversible perturbation that leaves the invariant distribution fixed. Indeed, our previous choice of irreversible perturbation remains valid for this system, that is, adding $\gamma (\theta ) = \mathbf {J}\nabla \log \pi (\theta )$ for a constant skew-symmetric matrix $\mathbf {J}$ to the drift term of (5) will preserve the invariant density. This choice yields the following SDE:

7 $$\begin{aligned} \text {d}\theta (t)&= \left[ (\beta \mathbf {B}(\theta (t))+\mathbf {J}) \nabla \log \pi (\theta (t)) + \beta \nabla \cdot \mathbf {B}(\theta (t)) \right] \text {d}t \nonumber \\&\quad + \sqrt{2\beta \mathbf {B}(\theta )} \text {d}W(t) \end{aligned}$$

We refer to this system as Riemannian manifold Langevin with an additive irreversible perturbation (RMIrr). This choice, however, does not take into account the relevant features that the reversible perturbation may provide when constructing an irreversible perturbation.

The reversible perturbation leads to a positive definite matrix (a metric, in the terminology of Riemannian geometry) that is state-dependent. In contrast, the skew-symmetric matrix $\mathbf {J}$ is fixed in the irreversible perturbation. The skew-symmetric matrix need not be constant, however, as an irreversible perturbation $\gamma (\theta )$ only needs to satisfy $\nabla \cdot (\gamma (\theta ) \pi (\theta ) )= 0$. In fact, if $\gamma (\theta ) = \mathbf {C}(\theta ) \nabla \log \pi (\theta ) + \nabla \cdot \mathbf {C}(\theta )$ for $\mathbf {C}(\theta ) = -\mathbf {C}(\theta )^T$, then this irreversible perturbation will also leave the invariant density intact. Noting that $\mathbf {C}_{ii}(\theta ) = 0$ and that $\mathbf {C}_{ij} = -\mathbf {C}_{ji}$, observe that

$$\begin{aligned} \nabla \cdot ( \gamma (\theta ) \pi (\theta ))&= \nabla \cdot ( \mathbf {C}(\theta ) \nabla \pi (\theta ) +(\nabla \cdot \mathbf {C}(\theta ) ) \pi (\theta ) ) \\&= \sum _{i,j = 1}^d \frac{\partial \mathbf {C}_{ij}(\theta )}{\partial \theta _i} \frac{\partial \pi (\theta )}{\partial \theta _j} + \mathbf {C}_{ij}(\theta ) \frac{\partial ^2 \pi (\theta )}{\partial \theta _i \partial \theta _j}\\&\quad + \frac{\partial ^2 \mathbf {C}_{ij}(\theta )}{\partial \theta _i\partial \theta _j} \pi (\theta ) + \frac{\partial \mathbf {C}_{ij}(\theta )}{\partial \theta _j} \frac{\partial \pi (\theta )}{\partial \theta _i} \\&=\sum _{i>j, i = 1}^d \frac{\partial \mathbf {C}_{ij}(\theta )}{\partial \theta _i} \frac{\partial \pi (\theta )}{\partial \theta _j} + \mathbf {C}_{ij}(\theta ) \frac{\partial ^2 \pi (\theta )}{\partial \theta _i \partial \theta _j}\\&\quad + \frac{\partial ^2 \mathbf {C}_{ij}(\theta )}{\partial \theta _i\partial \theta _j} \pi (\theta ) + \frac{\partial \mathbf {C}_{ij}(\theta )}{\partial \theta _j} \frac{\partial \pi (\theta )}{\partial \theta _j} \\&\quad + \frac{\partial \mathbf {C}_{ji}(\theta )}{\partial \theta _j} \frac{\partial \pi (\theta )}{\partial \theta _i} + \mathbf {C}_{ji}(\theta ) \frac{\partial ^2 \pi (\theta )}{\partial \theta _j\partial \theta _i}\\&\quad + \frac{\partial ^2 \mathbf {C}_{ji}(\theta )}{\partial \theta _j\partial \theta _i} \pi (\theta ) + \frac{\partial \mathbf {C}_{ji}(\theta )}{\partial \theta _i} \frac{\partial \pi (\theta )}{\partial \theta _i} \\&= \sum _{i>j, i = 1}^d \frac{\partial \mathbf {C}_{ij}(\theta )}{\partial \theta _i} \frac{\partial \pi (\theta )}{\partial \theta _j} + \mathbf {C}_{ij}(\theta ) \frac{\partial ^2 \pi (\theta )}{\partial \theta _i \partial \theta _j}\\&\quad + \frac{\partial ^2 \mathbf {C}_{ij}(\theta )}{\partial \theta _i\partial \theta _j} \pi (\theta ) + \frac{\partial \mathbf {C}_{ij}(\theta )}{\partial \theta _j} \frac{\partial \pi (\theta )}{\partial \theta _i} \\&\quad - \frac{\partial \mathbf {C}_{ij}(\theta )}{\partial \theta _j} \frac{\partial \pi (\theta )}{\partial \theta _i} - \mathbf {C}_{ij}(\theta ) \frac{\partial ^2 \pi (\theta )}{\partial \theta _j\partial \theta _i}\\&\quad -\frac{\partial ^2 \mathbf {C}_{ij}(\theta )}{\partial \theta _j\partial \theta _i} \pi (\theta ) - \frac{\partial \mathbf {C}_{ij}(\theta )}{\partial \theta _i} \frac{\partial \pi (\theta )}{\partial \theta _j}= 0. \end{aligned}$$

We seek an irreversible perturbation that takes the reversible perturbation into account, with the possibility that $\mathbf {C}(\theta )$ is not a constant matrix, and investigate if it leads to any performance improvements of the long term average estimator. Note that in the literature, the above condition $\nabla \cdot (\gamma \pi )=0$ is typically rewritten into the following sufficient conditions: $\nabla \cdot \gamma (\theta ) = 0$ and $\gamma (\theta ) \cdot \nabla \pi (\theta ) = 0$ (Rey-Bellet and Spiliopoulos 2016). One can check, however, that when $\mathbf {C}$ is not constant, these conditions are not met, yet $\gamma (\theta )$ is still a valid irreversible perturbation. A simple choice of $\mathbf {C}(\theta )$ that incorporates $\mathbf {B}(\theta )$ is

8 $$\begin{aligned} \mathbf {C}(\theta ) = \frac{1}{2}\mathbf {J}\mathbf {B}(\theta ) + \frac{1}{2}\mathbf {B}(\theta ) \mathbf {J}\, , \end{aligned}$$

where $\mathbf {J}$ is a constant skew-symmetric matrix. The $\frac{1}{2}$ factor is introduced so that if $\mathbf {B}(\theta ) = \mathbf {I}$, i.e., if there is no reversible perturbation, then this perturbation reverts to the standard irreversible perturbation (Irr). We arrive at the following system:

9 $$\begin{aligned} \text {d}\theta (t)= & {} \left[ (\beta \mathbf {B}(\theta (t)) + \mathbf {C}(\theta (t))) \nabla \log \pi (\theta (t)) \right. \nonumber \\&\left. + \nabla \cdot (\beta \mathbf {B}(\theta (t)) + \mathbf {C}(\theta (t)) \right] \text {d}t + \sqrt{2\beta \mathbf {B}(\theta (t))} \text {d}W(t).\nonumber \\ \end{aligned}$$

We call this choice of perturbation the *geometry-informed irreversible perturbation* (GiIrr). Indeed, while there are infinitely many valid choices for $\mathbf {C}(\theta )$, we will investigate the choice in (8) in the numerical examples. Since we will have already explicitly constructed $\mathbf {B}(\theta )$ and $\mathbf {J}$ for the other systems, the additional computational cost of computing their product will be marginal. Furthermore, as mentioned earlier, this choice reduces to Irr when $\mathbf {B}(\theta ) = \mathbf {I}$.

One may wonder when does GiIrr result in improved performance over standard irreversible perturbations such as in Equation 7. Based on the numerical results and intuition, we will argue that GiIrr results in better performance if the underlying reversible perturbation already improves the sampling. Namely, if one knows that RMLD leads to improved sampling on a given problem, then employing GiIrr is expected to improve sampling even further. As we mentioned earlier, the choice of GiIrr that is made in this paper is not unique, and a further investigation of its theoretical properties is left for future work; see also the discussion in the Section 4. The goal of this paper is to present this new class of irreversible perturbations and investigate it numerically in a number of representative computational studies.

### Stochastic gradient Langevin dynamics

In certain Bayesian inference problems, the data are conditionally independent of each other given the parameter value. Therefore, the likelihood model can often be factorized and the posterior density can be written as follows:

10 $$\begin{aligned} \pi (\theta ) \propto \pi _0(\theta ) \prod _{i = 1}^N \pi _i(X_i| \theta ) \end{aligned}$$

where $\pi (X_i|\theta )$ is the likelihood function for data point $X_i$. When the dataset is extremely large, i.e., when $N\gg 1$, however, ULA becomes exceedingly expensive as it requires repeatedly evaluating the likelihood over the entire dataset for each step of the trajectory. To mitigate this challenge, the stochastic gradient Langevin dynamics was presented to reduce the computational cost of evaluating the posterior density by only evaluating the likelihood over *subsets* of the data at each step. The true likelihood is estimated based on the likelihood function evaluated at the subsampled data (Welling and Teh 2011). Specifically, the gradient is estimated using a stochastic gradient

11 $$\begin{aligned} \nabla \log \pi (\theta |X)\approx & {} \widehat{\nabla \log \pi (\theta |X)}\nonumber \\= & {} \log \pi _0(\theta ) + \frac{N}{n} \sum _{i = 1}^n \log \pi (X_{\tau _i}|\theta ) \end{aligned}$$

where $\tau$ is a random subset of $\{1,\ldots , N\}$ of size *n* drawn with or without replacement. Depending on the choice of *n*, this approach cuts down on the computational costs dramatically with some additional variance incurred by the random subsampling of the data. The original version of this algorithm made the step size variable, approaching zero as the number of steps taken *K* became large. SGLD applied with a variable and shrinking step size was proven to be consistent: that is, the invariant distribution of the discretized system is equivalent to that of the continuous system (Teh et al. 2016). Having a decreasing step size counteracts the cost savings provided by computing the stochastic gradient, and therefore a version where the step size is fixed was presented in Vollmer et al. (2016), where theoretical characterizations of the asymptotic and finite-time bias and variance are also developed. In most of our numerical results, we use stochastic gradient version of the Langevin algorithm with fixed step size to demonstrate that SGLD can be used together with irreversible perturbations.

## Numerical examples

In the following examples, we always apply the stochastic gradient version of each Langevin system unless otherwise stated. We fix $\beta = 1/2$ for all examples. The efficacy of the GiIrr perturbation does not change whether or not the stochastic gradient is used. We illustrate this explicitly in Section 3.4, where we report the results of all perturbations both with and without the stochastic gradient, for comparison.

### Evaluating sample quality

Here we discuss the measures we use to evaluate sample quality for each Langevin sampler. For each example, we estimate the bias, variance, mean-squared error, and asymptotic variance of the estimators of the expectations of two observables: $\phi _1(\theta ) = \sum _{l = 1}^d \theta ^{(l)}$ and $\phi _2(\theta ) = \sum _{l = 1}^d \left| \theta ^{(l)}\right| ^2$, where $\theta ^{(l)}$ denotes the *l*th component of $\theta$. Let $\bar{\phi }^K = \frac{1}{K}\sum _{k = 0}^{K-1} \phi (\theta _k)$ denote the estimator of ${\mathbb {E}}_\pi [\phi (\theta )]$ obtained with a chain of length *K*.

12 $$\begin{aligned} \text {Bias}(\bar{\phi }^K)&= {\mathbb {E}}\left[ \bar{\phi }^K \right] - {\mathbb {E}}_\pi \left[ \phi (\theta )\right] \nonumber \\&= {\mathbb {E}}\left[ \frac{1}{K}\sum _{k = 0}^{K-1} \phi (\theta _k)\right] -{\mathbb {E}}_\pi \left[ \phi (\theta )\right] \nonumber \\&\approx \frac{1}{M}\sum _{i=1}^{M} \left[ \frac{1}{K}\sum _{k = 0}^{K-1} \phi ([\theta _k]_{i}) \right] -{\mathbb {E}}_\pi \left[ \phi (\theta )\right] , \end{aligned}$$

where $[\theta _k]_{i}$ is the state of the $i^{th}$ chain at iteration *k*. Here, we estimate ${\mathbb {E}}_\pi \left[ \phi (\theta ) \right]$ by applying the unadjusted Langevin algorithm with a very long simulated trajectory and small discretization step. The expected value of the estimator is computed by averaging over $M=1000$ independent chains for the examples in Sections 3.2 and 3.3, and $M = 100$ independent chains for the examples in Sections 3.4 and 3.5. The variance of each estimator is defined and estimated as follows

13 $$\begin{aligned} \text {Var}(\bar{\phi }^K)&= {\mathbb {E}}\left[ (\bar{\phi }^K)^2\right] -\left( {\mathbb {E}}\left[ \bar{\phi }^K \right] \right) ^2 \nonumber \\&\approx \frac{1}{M}\sum _{i=1}^{M}\left( \frac{1}{K}\sum _{k = 0}^{K-1} \phi ([\theta _k]_{i}) \right) ^2 \nonumber \\&\quad - \left( \frac{1}{M}\sum _{i=1}^{M}\left[ \frac{1}{K}\sum _{k = 0 }^{K-1} \phi ([\theta _k]_{i})\right] \right) ^2. \end{aligned}$$

The mean-squared error (MSE) of each estimator $\bar{\phi }^K$ follows analogously.

We also evaluate the asymptotic variance of the estimator of each observable, defined as

14 $$\begin{aligned} \sigma ^2(\phi ) = \lim _{t\rightarrow \infty } t \, \text {Var}\left( \frac{1}{t} \int _0^t \phi (\theta _t) \text {d}t\right) \approx \lim _{K\rightarrow \infty } Kh \text {Var}\left( \bar{\phi }^K \right) . \end{aligned}$$

To compute these asymptotic variances, we use the batch means method in Asmussen and Glynn (2007). After the burn-in period, we evaluate the observable over each chain. Each observable chain is then batched into twenty separate chains, and their means are evaluated. The asymptotic variance is estimated by computing the empirical variance of those means and then multiplying by the length of each of the subsampled trajectories.

In addition to measuring the performance of estimators of specific observables, for each sampler we also evaluate overall sample quality by computing the recently-proposed kernelized Stein discrepancy (KSD) (Gorham and Mackey 2017). The KSD is a computable expression that can approximate certain integral probability metrics (IPMs) for a certain class of functions defined through the action of the Stein operator on a reproducing kernel Hilbert space. Let $\hat{\pi }_K = \frac{1}{K}\sum _{k = 0}^{K-1} \delta _{\theta _k}$ be the empirical approximation to $\pi$ based on samples $\{\theta _k\}_{k=0}^{K-1}$ produced by some Langevin algorithm. The IPM is defined as

15 $$\begin{aligned} d_{{\mathcal {H}}}(\hat{\pi }_K,\pi ) := \sup _{h\in {\mathcal {H}}} \left| {\mathbb {E}}_{\hat{\pi }_K}[h(Z)] - {\mathbb {E}}_{\pi }[h(X)] \right| , \end{aligned}$$

for some function space ${\mathcal {H}}$, where $h\in {\mathcal {H}}$ are functions from ${\mathbb {R}}^d$ to ${\mathbb {R}}$, and $Z\sim \hat{\pi }_K$ and $X\sim \pi$ are random variables. When ${\mathcal {H}}$ is large enough, $d_{{\mathcal {H}}}(\hat{\pi }_K,\pi )\rightarrow 0$ holds only if $\hat{\pi }_K\rightarrow \pi$ in distribution; see Gorham and Mackey (2017) and the references therein. This result implies that a better empirical approximation (i.e., better-quality samples) corresponds with a lower IPM value. Practically computing the IPM directly is intractable as it requires exactly knowing expectations with respect to the target distribution $\pi$ (which is, after all, our original goal in sampling). By judiciously choosing the function space ${\mathcal {H}}$, however, we can estimate the IPM by computing the KSD, using only the samples that comprise the empirical distribution $\hat{\pi }$.

As the expectation with respect to the target distribution $\pi$ is intractable to compute, one seeks an ${\mathcal {H}}$ such that for all $h\in {\mathcal {H}}$, ${\mathbb {E}}_\pi [h(X)] = 0$. Such a function space is found through *Stein’s identity*, which states that ${\mathbb {E}}_\pi [{\mathcal {A}}g(X)] = 0$ for all $g:{\mathbb {R}}^d\rightarrow {\mathbb {R}}^d$ in some function space ${\mathcal {G}}$, where ${\mathcal {A}}$ is the Stein operator,

$$\begin{aligned} {\mathcal {A}}g(x)=\frac{1}{\pi (x)}\nabla \cdot \left( \pi (x)g(x)\right) = g(x)\cdot \nabla \log \pi (x) + \nabla \cdot g(x), \end{aligned}$$

and $\nabla \cdot$ denotes the divergence operator. The space ${\mathcal {G}}$ is defined by specifying a reproducing kernel Hilbert space (RKHS) ${\mathcal {R}}_r$ of functions from ${\mathbb {R}}^d$ to ${\mathbb {R}}$, with a user-defined kernel *r*(*x*, *y*) that is twice-continuously differentiable (Gorham and Mackey 2017; Izzatullah et al. 2020). Each function $g \in {\mathcal {G}}$ is made up of components $g_j\in {\mathcal {R}}_r$, for $j = 1,\ldots ,d$, such that the vector $(\Vert g_1 \Vert _{{\mathcal {R}}_r},\ldots ,\Vert g_d \Vert _{{\mathcal {R}}_r})$ has unit norm in the dual space $\ell ^2$, where $\Vert \cdot \Vert _{{\mathcal {R}}_r}$ is the norm of the RKHS (Gorham and Mackey 2017). Hence, if we set $h = {\mathcal {A}} g$ for $g \in {\mathcal {G}}$, then ${\mathbb {E}}_\pi [h(X)] = 0$. Proposition 1 in Gorham and Mackey (2017) demonstrates that such a ${\mathcal {G}}$ is an appropriate domain for ${\mathcal {A}}$ so that one indeed has ${\mathbb {E}}_\pi [{\mathcal {A}}g(X)] = 0$ for all $g \in {\mathcal {G}}$. Then the space ${\mathcal {H}}$ is defined as ${\mathcal {H}} = {\mathcal {A}} {\mathcal {G}}$, i.e., the space of functions resulting from the Stein operator applied to functions in ${\mathcal {G}}$. With this ${\mathcal {H}}$, we define the corresponding KSD of a measure $\mu$ as ${\mathcal {S}}(\mu )=d_{{\mathcal {H}}}(\mu ,\pi )=\sup _{h\in {\mathcal {H}}}\left| {\mathbb {E}}_{\mu }[h(X)] \right|$.

Proposition 2 in Gorham and Mackey (2017) shows that the KSD admits a closed form. Indeed, for any $j=1,\ldots , d$ and letting $b_{j}(x)=\partial _{x_{j}}\log \pi (x)$, define the Stein kernel

16 $$\begin{aligned} r_0^j(x,y)&= b_j(x)b_j(y) r(x,y) + b_j(x) \nabla _{y_j}r(x,y)\nonumber \\&\quad +b_j(y) \nabla _{x_j}r(x,y) + \nabla _{x_j}\nabla _{y_j} r(x,y). \end{aligned}$$

Then, by Proposition 2 of Gorham and Mackey (2017), if $\sum _{j=1}^{d}{\mathbb {E}}_{\mu }\left[ \sqrt{r_0^j(X,X)}\right] <\infty$, we have the equality ${\mathcal {S}}(\mu ) = \Vert w\Vert _2$, where for $j=1,\ldots ,d$ we have

$$\begin{aligned} w_j = \sqrt{{\mathbb {E}}_{\mu \times \mu }\left[ r_0^j(X,\tilde{X})\right] }, \ \ X,\tilde{X} {\mathop {\sim }\limits ^{\text {\tiny i.i.d.}}} \mu . \end{aligned}$$

Also, Gorham and Mackey (2017) emphasizes the importance of choosing the kernel *r*(*x*, *y*) carefully so that convergence of the KSD to zero (for a sequence of empirical distributions) implies convergence in distribution to the target measure. As suggested by the results of Gorham and Mackey (2017), we use the inverse multiquadric kernel $r(x,y) = (c^2 + \Vert x-y\Vert _2^2)^\beta$ with $\beta = -1/2$ and $c = 1$. This choice has also been used in Izzatullah et al. (2020).

In our examples, $\mu = \hat{\pi }_K$ is a discrete distribution, so the KSD can be computed by evaluating the kernels over all pairs of sample points (Gorham and Mackey 2017). Namely, if $\mu = \hat{\pi }_K = \frac{1}{K} \sum _{k = 0}^{K-1} \delta _{\theta _k}$, then

17 $$\begin{aligned} w_j = \sqrt{\sum _{k,k' = 1}^K r_0^j(x_k,x_{k'}) } \, . \end{aligned}$$

We use the KSD to evaluate the quality of every Langevin sampler—effectively, sampler performance over a large class of observables—to complement our bias/variance/MSE computations for particular choices of observable. For each example below and for each Langevin sampler, we compute the KSD for 25 independent chains. The mean KSDs of the chains for each sampler are then plotted as a function of the number of steps of the chain. We comment on the quality of each sampler given these KSD plots. We refer the reader to Gorham et al. (2019), Gorham and Mackey (2015, 2017), Izzatullah et al. (2020) for further details on these choices and for further literature review.

### Linear Gaussian example

Suppose we have data $\{X_i\}_{i = 1}^N \subset {\mathbb {R}}^d$ generated from a multivariate normal distribution with mean $\theta \in {\mathbb {R}}^d$ and known precision matrix ${\varvec{\Gamma }}_X \in {\mathbb {R}}^{d\times d}$. From the data, we infer the value of $\theta$. Endow $\theta$ with a normal prior with mean zero and precision ${\varvec{\Gamma }}_\theta \in {\mathbb {R}}^{d\times d}$. Then the posterior distribution is Gaussian with mean and precision

18 $$\begin{aligned} \mu _p = ({\varvec{\Gamma }}_\theta + N {\varvec{\Gamma }}_X)^{-1}{\varvec{\Gamma }}_X \sum _{i = 1}^N X_i\,\, \text {and} \,\, {\varvec{\Gamma }}_p = ({\varvec{\Gamma }}_\theta +N {\varvec{\Gamma }}_X), \end{aligned}$$

respectively. The Euler-Maruyama discretization with constant step size *h* applied to the corresponding Langevin dynamics is

19 $$\begin{aligned} \theta _{k+1} = \left( \mathbf {I}- \bar{\mathbf {A}}h \right) \theta _k +\bar{\mathbf {D}}_k h + \sqrt{h}\xi _k \end{aligned}$$

where

$$\begin{aligned} \bar{\mathbf {A}} = \frac{1}{2} ({\varvec{\Gamma }}_\theta + N {\varvec{\Gamma }}_X), \,\,\,\, \bar{\mathbf {D}}_k = \frac{1}{2}{\varvec{\Gamma }}_X \sum _{i = 1}^N X_i, \,\,\,\, \xi _k \sim {\mathcal {N}}(0,\mathbf {I}). \end{aligned}$$

Using stochastic gradients yields the same recurrence above except with

20 $$\begin{aligned} \bar{\mathbf {D}}_k = \frac{1}{2}{\varvec{\Gamma }}_X \frac{N}{n} \sum _{i = 1}^n X_{\tau ^k_i} \end{aligned}$$

where $n \le N$ and $\tau ^k_i\in \{1,\ldots , N\}$ is randomly sampled (with or without replacement) (Welling and Teh 2011). Expectations with respect to the posterior are approximated by an long term average of the observable over the course of a trajectory. It has been shown that despite subsampling the data at each step in the dynamics, this estimator has comparable performance as the estimator produced by the regular Langevin dynamics with the full likelihood or MALA (Welling and Teh 2011; Vollmer et al. 2016).

Now, we consider the case where the dynamics are perturbed by an irreversible term that preserves the invariant distribution of the dynamics. We demonstrate that this leads to a lower MSE than standard SGLD or Langevin dynamics. In this case, we replace $\bar{\mathbf {A}}$ and $\bar{\mathbf {D}}_k$ with ${\mathbf {A}}$ and ${\mathbf {D}}_k$, which are

21 $$\begin{aligned} {\mathbf {A}} = \frac{1}{2}(\mathbf {I}+\mathbf {J})({\varvec{\Gamma }}_\theta + N{\varvec{\Gamma }}_X), \,\,\,\, {\mathbf {D}}_k = \frac{1}{2}(\mathbf {I}+ \mathbf {J}){\varvec{\Gamma }}_X \frac{N}{n} \sum _{i = 1}^n X_{\tau _i^k}. \end{aligned}$$

and $\mathbf {J}$ is a skew-symmetric matrix.

**Table 1:** Summary of the five SDEs that share the same invariant density $\pi (\theta )$. Stochastic gradients can be considered instead of the deterministic gradients. All systems are of the form $\text {d}\theta _t = b(\theta _t) \text {d}t + \sigma (\theta _t) \text {d}W_t$. The term $\beta$ denotes the temperature

	$b(\theta )$	$\sigma (\theta )$
LD	$\beta \nabla \log \pi (\theta )$	$\sqrt{2\beta } \mathbf {I}$
RM	$\beta \mathbf {B}(\theta )\nabla \log \pi (\theta )) + \beta \nabla \cdot \mathbf {B}(\theta )$	$\sqrt{2\beta \mathbf {B}(\theta )}$
Irr	$(\beta \mathbf {I}+ \mathbf {J}) \nabla \log \pi (\theta )$	$\sqrt{2\beta } \mathbf {I}$
RMIrr	$(\beta \mathbf {B}(\theta ) + \mathbf {J})\nabla \log \pi (\theta ) +\beta \nabla \cdot \mathbf {B}(\theta )$	$\sqrt{2\beta \mathbf {B}(\theta )}$
GiIrr	$(\beta \mathbf {B}(\theta ) + \frac{1}{2}\mathbf {J}\mathbf {B}(\theta ) + \frac{1}{2}\mathbf {B}(\theta ) \mathbf {J})\nabla \log \pi (\theta ) + \nabla \cdot (\beta \mathbf {B}(\theta ) + \frac{1}{2}\mathbf {J}\mathbf {B}(\theta ) + \frac{1}{2}\mathbf {B}(\theta ) \mathbf {J})$	$\sqrt{2\beta \mathbf {B}(\theta )}$

**Fig. 1:**
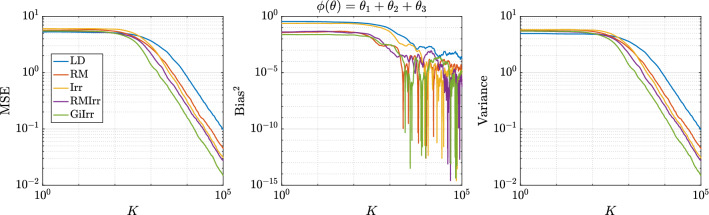
MSE of the running average for the first moment. Stochastic gradients are computed

**Fig. 2:**
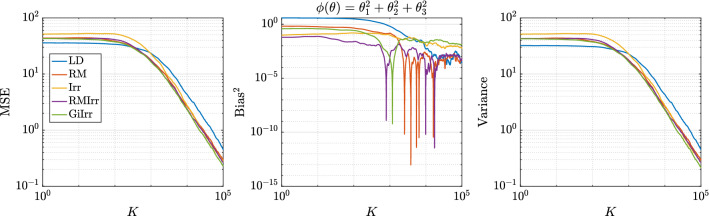
MSE of the running average for the second moment. Stochastic gradients are computed

For the numerical experiments, we choose $d = 3$, $N = 10$, where the mini-batches are of size $n = 2$. The initial condition is the zero vector. We have ${\varvec{\Gamma }}_X = 0.25\mathbf {I}$, ${\varvec{\Gamma }}_\theta$ is a precision matrix with eigenvalues 0.2, 0.01, 0.05 and eigenvectors that are randomly generated, and $h = 0.005$. Note that these matrices were chosen so that the resulting reversible perturbation has eigenvalues greater than one. To construct the perturbations, we choose $\mathbf {B}= {\varvec{\Gamma }}_p^{-1}$ and $\mathbf {J}$ to be

22 $$\begin{aligned} \mathbf {J}= \delta \begin{bmatrix} 0 &{} 1 &{} 1 \\ -1 &{} 0 &{} 1 \\ -1 &{} -1 &{} 0 \end{bmatrix} \end{aligned}$$

for $\delta \in {\mathbb {R}}$. We consider the five different SDE systems presented in Table 1 and investigate how the MSE, bias, and variance differs for each case. For this example, since a constant metric is used, the geometry-informed irreversible perturbation simply produces a different constant skew-symmetric matrix than the other irreversible perturbations. Each system is simulated for $K = 10^5$ steps with step size $h = 5\times 10^{-3}$. In Figures 1 and 2, we plot the MSE of the running average for each case when the observables are the sums of the first and second moments. In Table 2, we report the asymptotic variance of the estimator for each system. We see that irreversible perturbations definitely improve the performance of the estimators, although the improvement provided by the geometry-informed irreversible perturbation seems marginal over RMIrr when estimating the second moments.

When the reversible perturbation is chosen such that the drift matrix is exactly the identity (for example, when the matrix is chosen to be the covariance matrix of the posterior), additional irreversibility cannot widen the spectral gap of the system. This fact can be deduced from the results of Lelievre et al. (2013). The improved performance of the geometry-informed irreversible perturbation is mostly due to the fact that the norm of the corresponding skew-symmetric matrix is greater than that of simple irreversibility. Even though one can scale the skew-symmetric matrix for the other two cases to observe similar performance as geometry-informed irreversibility, GiIrr accomplishes that in a more systematic way.

**Fig. 3:**
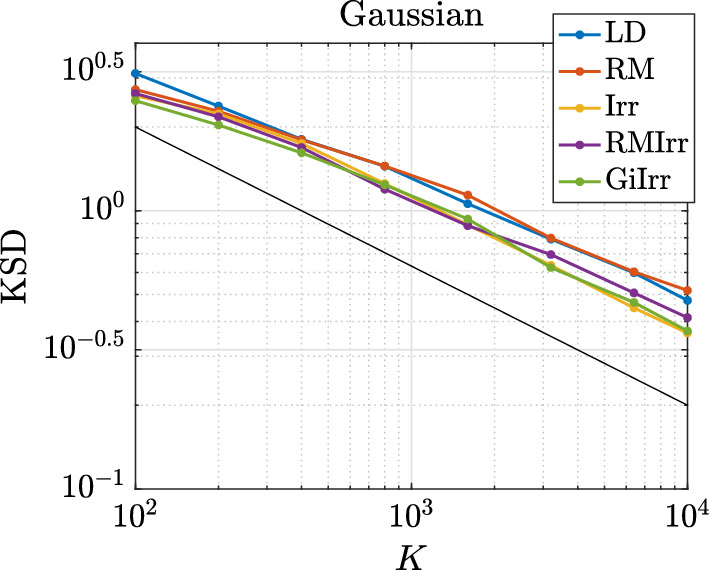
Kernelized Stein discrepancy plot for the Gaussian example. Black line has slope $-1/2$, which denotes the expected convergence rate

In Figure 3 we plot the kernelized Stein discrepancy (KSD) for the linear–Gaussian example. We see that irreversible perturbations typically have smaller KSD compared to reversible perturbations and that in all cases the theoretical slope of $K^{-1/2}$ (see Liu et al. (2016)) is achieved.

#### Remark 3.1

Note that in Figure 3 and in all subsequent KSD-related figures, *K* ranges up to $10^4$, rather than up to $10^{5}$ or higher (as in the bias/variance/MSE plots, e.g., Figures 1 and 2 and analogous figures in subsequent examples). The reason is that KSD is expensive to compute, and we find that evaluating it up to $K=10^4$ is sufficient to draw conclusions.

#### Remark 3.2

While it is known that irreversible perturbations can, at worst, maintain the same performance as standard Langevin in the continuous-time setting (Rey-Bellet and Spiliopoulos 2016), when considering discretization and in borderline cases (i.e., when one does not expect much or any improvement in continuous time), irreversibility may actually harm the performance of the estimator as it introduces additional stiffness into the system without resulting in faster convergence to the invariant density. A detailed exploration of this effect is presented in Appendix A, in which we compute the bias and variance of the long term average estimator for a simple linear Gaussian problem where the posterior precision is a scalar multiple of the identity matrix. As further discussed in Remark A.1, in this case, the irreversible perturbation is not expected to lead to improvement in the sampling properties from the equilibrium. Hence, the stiffness induced upon discretization has a more profound impact on the practical performance of the irreversible perturbation.

**Table 2:** Asymptotic variance estimates for the linear Gaussian example

	${\mathbb {E}}[\text {AVar}_{\phi _1}]$	$\text {Std}[\text {AVar}_{\phi _1}]$	${\mathbb {E}}[\text {AVar}_{\phi _2}]$	$\text {Std}[\text {AVar}_{\phi _2}]$
LD	37.75	11.94	209.4	84.38
RM	20.09	6.420	132.8	49.21
Irr	15.72	5.008	135.4	47.91
RMIrr	12.36	3.937	115.9	40.10
GiIrr	7.444	2.336	$\mathbf {103.7}$	36.78

In the current numerical study, the posterior precision is diagonal, but not a scalar multiple of the identity matrix. The eigenvalues of the resulting drift matrix are therefore distinct, and by the theory in Lelievre et al. (2013), irreversible perturbations are able to reduce the spectral gap and result in improved performance. This is in contrast with the example studied in Appendix A. In the example of the appendix, the drift matrix is taken to be proportional to the identity matrix, and as explained in Remark A.1, the spectral gap therefore cannot be widened. In that case, irreversibility leads to increased stiffness of the system, which then leads to increased bias and variance in the resulting estimator.

### Parameters of a normal distribution

This example is identical to that used in Girolami and Calderhead (2011, Section 5) to demonstrate the performance of RMLD. Given a dataset of ${\mathbb {R}}$-valued data $\mathbf {X} = \{X_i\}_{i = 1}^N \sim {\mathcal {N}}(\mu ,\sigma ^2)$, we infer the parameters $\mu , \sigma$. To be clear, in this example the state is $\theta =[\mu ,\sigma ]^T$. The prior on $\mu ,\sigma$ is chosen to be flat (and, therefore, improper). The log-posterior is

23 $$\begin{aligned} \log p(\mu ,\sigma |\mathbf {X}) = \frac{N}{2} \log 2\pi - N \log \sigma - \sum _{i = 1}^N \frac{(X_i-\mu )^2}{2\sigma ^2}. \end{aligned}$$

The gradient is

24 $$\begin{aligned} \nabla \log p(\mu ,\sigma |\mathbf {X}) = \begin{bmatrix} m_1(\mu )/\sigma ^2 \\ -N/\sigma + m_2(\mu )/\sigma ^3 \end{bmatrix} \end{aligned}$$

where $m_1(\mu ) = \sum _{i = 1}^N (X_i - \mu )$, and $m_2(\mu ) = \sum _{i = 1}^N (X_i-\mu )^2$. In Girolami and Calderhead (2011), the authors propose using the geometry of the manifold defined by the parameter space of the posterior distribution to accelerate the resulting Metropolis-adjusted Langevin algorithm. The authors in Girolami and Calderhead (2011) suggest using the expected Fisher information matrix to define the Riemannian metric, which in the context of reversible diffusions (Rey-Bellet and Spiliopoulos 2016), is equivalent to choosing $\mathbf {B}(\mu ,\sigma )$ to be the inverse of the sum of the expected Fisher information matrix and the negative Hessian of the log-prior. Straightforward computations yield

25 $$\begin{aligned} \mathbf {B}= \frac{\sigma ^2}{N} \begin{bmatrix} 1 &{} 0 \\ 0 &{} 1/2 \end{bmatrix}, \;\; \sqrt{\mathbf {B}} = \frac{\sigma }{\sqrt{N}} \begin{bmatrix} 1 &{} 0 \\ 0 &{} 1/\sqrt{2} \end{bmatrix},\;\; \nabla \cdot \mathbf {B}= \begin{bmatrix} 0 \\ \sigma /N \end{bmatrix}. \end{aligned}$$

As for the geometry-informed irreversible perturbation, let $$\mathbf {J}= \delta \begin{bmatrix}0 &{} 1 \\ -1 &{} 0 \end{bmatrix}$$, for $\delta \in {\mathbb {R}}$. Then the relevant quantities are

26 $$\begin{aligned} \frac{1}{2}\mathbf {J}\mathbf {B}+ \frac{1}{2}\mathbf {B}\mathbf {J}= \frac{3 \sigma ^2}{4N}\mathbf {J}, \;\; \frac{1}{2}\nabla \cdot (\mathbf {J}\mathbf {B}+ \mathbf {B}\mathbf {J}) = \frac{3\delta \sigma }{2N}\begin{bmatrix} 1 \\ 0 \end{bmatrix}. \end{aligned}$$

**Fig. 4:**
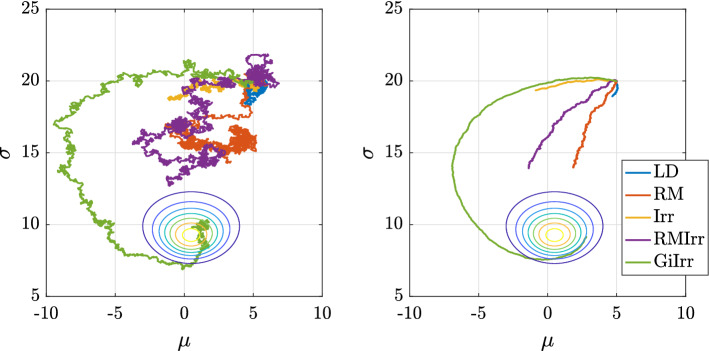
Trajectory burn-in: each trajectory is run for $T = 2.5$. Left: single trajectories, right: mean paths. The gradients are computed exactly here

**Fig. 5:**
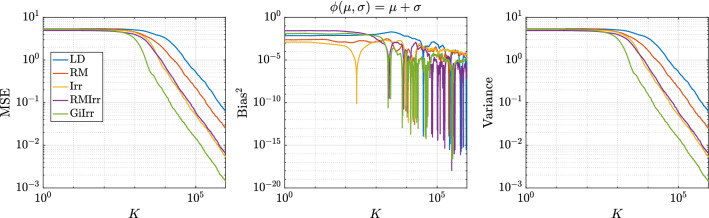
Observable: $\phi _1(\mu ,\sigma ) = \mu +\sigma$, $\delta = 2$. Stochastic gradients are computed

**Fig. 6:**
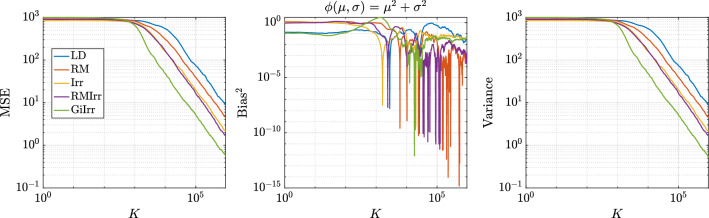
Observable: $\phi _2(\mu ,\sigma ) = \mu ^2 + \sigma ^2$, $\delta = 2$. Stochastic gradients are computed

**Fig. 7:**
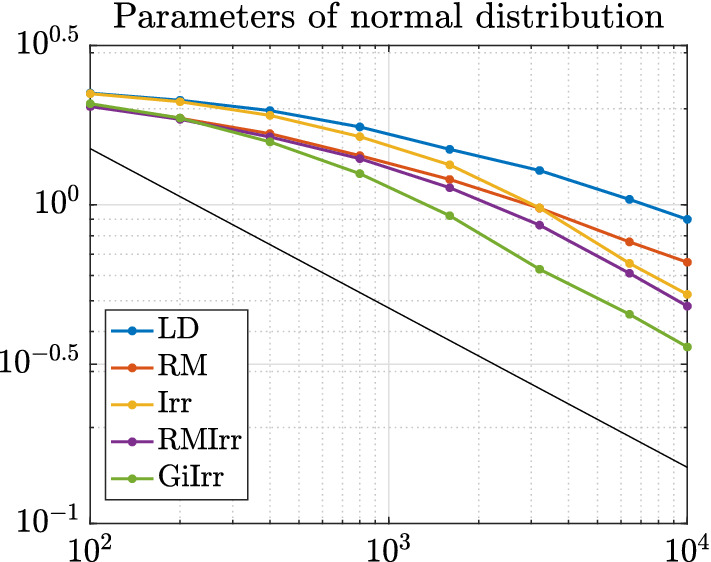
Kernelized Stein discrepancy plot for the parameters of a normal distribution example. Black line has slope $-1/2$, which denotes the expected convergence rate

In the experiments, we have $N = 30$, $h = 10^{-3}$, $\delta = 2$. and simulate $M = 1000$ independent trajectories of each system up to $T = 1000$ for a total of $K = 10^6$ steps. The initial condition is chosen to be $\mu = 5$ and $\sigma = 20$, which is consistent with the choice in Girolami and Calderhead (2011). The data are subsampled at a rate of $n = 6$ per stochastic gradient computation. Each trajectory is allotted a burn-in time of $T_b = 10$. The dataset is generated by drawing samples from a normal distribution with $\mu _{true} = 0$ and $\sigma _{true} = 10$. The observables we study are $\phi _1(\mu ,\sigma ) = \mu + \sigma$, and $\phi _2(\mu ,\sigma ) = \mu ^2 + \sigma ^2$. We plot the MSE, squared bias, and variance of resulting estimators for each observable in Figures 5 and 6. Moreover, in Table 3 we report the asymptotic variance of the estimators of each of the five systems. We plot the kernelized Stein discrepancy in Figure 7. Notice that the irreversibly perturbed systems reach the $K^{-1/2}$ convergence rate (see Liu et al. (2016)) faster than the reversibly perturbed system. The main takeaway is that an irreversible perturbation that is adapted to the existing reversible perturbation performs much better than if the irreversible perturbation were applied without regard to the underlying geometry. Notice that the reversible perturbation considered here still improves the performance of the long term average estimator despite the fact that $\mathbf {B}-\mathbf {I}$ is not positive definite on the state space. Indeed, while $\mathbf {B}-\mathbf {I}$ being positive definite is a sufficient condition to obtain improved performance, it is not a necessary one (Rey-Bellet and Spiliopoulos 2016). The reason for the reduced asymptotic variance we observed here is because the reversible perturbation $\mathbf {B}$ has eigenvalues larger than one where the bulk of the posterior distribution lies.

**Table 3:** Asymptotic variance estimates for the parameters of a normal distribution example. Stochastic gradients are employed

	${\mathbb {E}}[\text {AVar}_{\phi _1}]$	$\text {Std}[\text {AVar}_{\phi _1}]$	${\mathbb {E}}[\text {AVar}_{\phi _2}]$	$\text {Std}[\text {AVar}_{\phi _2}]$
LD	55.29	21.52	8332	4359
RM	20.63	6.019	4034	1378
Irr	5.791	2.638	2169	1072
RMIrr	6.512	2.226	1729	631.2
GiIrr	$\mathbf {1.400}$	0.4697	$\mathbf {479.4}$	170.8

Figure 4 show single and mean trajectories of the burn-in period of trajectories from each of the five systems. The plot shows that the geometry-informed irreversible perturbation is able to find the bulk of the distribution sooner than the other systems without incurring additional errors due to stiffness.

To show that the GiIrr perturbation is not intimately tied to the stochastic gradient, we also report the results for each system when the gradients are computed exactly in Table 4. We see that there is little meaningful difference in the results compared to when stochastic gradients are used.

In Figure 7 we plot the KSD for the parameters-of-a-normal-distribution example. We see that GiIrr yields lower KSD than all other perturbations.

**Table 4:** Asymptotic variance estimates for the parameters of a normal distribution example. The gradients are computed exactly

	${\mathbb {E}}[\text {AVar}_{\phi _1}]$	$\text {Std}[\text {AVar}_{\phi _1}]$	${\mathbb {E}}[\text {AVar}_{\phi _2}]$	$\text {Std}[\text {AVar}_{\phi _2}]$
LD (no SG)	48.51	17.53	7339	3707
RM (no SG)	20.91	6.445	3855	1406
Irr (no SG)	5.658	2.108	2265	1191
RMIrr (no SG)	6.276	2.075	1648	565.1
GiIrr (no SG)	$\mathbf {1.363}$	0.4223	$\mathbf {492.9}$	183.8

### Bayesian logistic regression

Next we consider Bayesian logistic regression. Given data $\{(\mathbf {x}_i,t_i)\}_{i = 1}^N$, where $\mathbf {x}_i \in {\mathbb {R}}^d$, and $t_i \in \{0,1\}$, we seek a logistic function, parameterized by weights $\mathbf {w}\in {\mathbb {R}}^d$, that best fits the data. The weights are obtained in a Bayesian fashion, in which we endow the weights with a prior and seek to characterize its posterior distribution via sampling. Define $\varphi (y)$ to be the logistic function $\varphi (y)= ({1+\exp (-y) })^{-1}$. The log-likelihood function is

27 $$\begin{aligned} l(\mathbf {w}) = \sum _{i = 1}^N t_i \mathbf {x}_i^T \mathbf {w}- \sum _{i = 1}^N \log (1 + \exp (\mathbf {x}_i^T \mathbf {w}) ). \end{aligned}$$

The prior for the weights is normally distributed with mean zero and covariance $\alpha ^{-1} \mathbf {I}$. The gradient of the log-posterior is

28 $$\begin{aligned} \nabla _{\mathbf {w}} \log \pi (\mathbf {w}| \mathbf {X}) = -\alpha \mathbf {w}+ \sum _{i = 1}^N t_i \mathbf {x}_i - \sum _{i = 1}^N \varphi (\mathbf {x}_i^T \mathbf {w}) \mathbf {x}_i. \end{aligned}$$

This term is used in the drift part of the Langevin dynamics that fully computes the gradient of the log-likelihood at every step. If the data are subsampled as in SGLD, we instead compute

29 $$\begin{aligned} \nabla _{\mathbf {w}}\log \tilde{\pi }(\mathbf {w}|\mathbf {X}) = -\alpha \mathbf {w}+ \frac{N}{n}\sum _{i = 1}^n t_{\tau _i} \mathbf {x}_{\tau _i} -\frac{N}{n}\sum _{i = 1} ^n \phi (\mathbf {x}_{\tau _i}^T\mathbf {w})\mathbf {x}_{\tau _i}. \end{aligned}$$

We use the german data set described in Gershman et al. (2012) for the numerical experiments. In this problem, there are 20 weight parameters to be learned. The training dataset is of size $N = 400$ and we choose to subsample at a rate of $n = 10$ per likelihood computation. The time step we choose is $h = 10^{-4}$ and $K = 4\times 10^{5}$ steps. The initial condition is chosen to be the zero vector. We generate the skew-symmetric matrix by constructing a lower triangular matrix with entries randomly drawn from $\{1,-1\}$ and then subtracting its transpose. The diagonal is then set to zero and the matrix is scaled to have norm one.

**Fig. 8:**
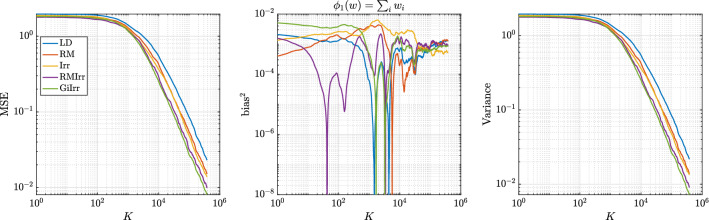
Observable: $\phi _1(w) = \sum _{i}w_{i}$. Bayesian logistic regression. Here, $d = 20$

**Fig. 9:**
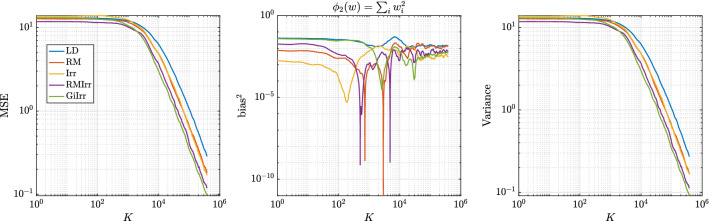
Observable: $\phi _2(w) = \sum _{i}w^{2}_{i}$. Bayesian logistic regression. Here, $d = 20$

As for the Riemannian manifold Langevin dynamics, in Girolami and Calderhead (2011) the authors use the expected Fisher information matrix plus the negative Hessian of the log-prior as the underlying metric, which in this case is equal to

30 $$\begin{aligned} \mathbf {G}(w) = \alpha ^{-1}\mathbf {I}+ \mathbf {X}\varvec{\Lambda }(w) \mathbf {X}^T \end{aligned}$$

where $\varvec{\Lambda }$ is a diagonal matrix with entries $\varvec{\Lambda }_{ii}(w) = (1-\varphi (\mathbf {x}_i^T w) ) \varphi (\mathbf {x}_i^Tw)$ and $\mathbf {x}_i$ is the *i*-th column of $\mathbf {X}$. The resulting reversible perturbation uses the inverse of $\mathbf {G}(w)$. This perturbation, however, does not lead to accelerated convergence to the invariant measure since the eigenvalues of $\mathbf {G}$ are large. This implies that the eigenvalues of $\mathbf {G}^{-1}$ are less than one and so $\mathbf {G}^{-1}(w) -\mathbf {I}$ is not positive definite, a condition that needs to be satisfied to guarantee accelerated convergence (Rey-Bellet and Spiliopoulos 2015). To alleviate this issue, we consider the reversible perturbation $\mathbf {B}(w) = \mathbf {I}+ \mathbf {G}^{-1}(w)$. This guarantees $\mathbf {B}(w)$ to be positive definite for all *w*, but the drawback is that computing the square root of $\mathbf {B}(w)$ requires explicitly computing or at least approximating the inverse of $\mathbf {G}(w)$ repeatedly in the simulation (and not just computing the action of the inverse). This additional computational cost is incurred for all examples that consider a geometry-informed perturbation, both reversible and irreversible. We show the result of this state-dependent perturbation in Figures 8 and 9 and report the asymptotic variance in Table 5. The geometry-informed irreversible perturbation does provide improvement over all other perturbations. We observe that the asymptotic variance is reduced by half over RM, with only little additional computational effort. Most of the computational cost of applying GiIrr is due to the evaluation of the reversible perturbation. Therefore we emphasize that if one is already applying the reversible perturbation to the Langevin dynamics, the marginal cost of applying the GiIrr perturbation is negligible.

**Table 5:** Asymptotic variance estimates for the Bayesian logistic regression example with a state-dependent metric

	${\mathbb {E}}[\text {AVar}_{\phi _1}]$	$\text {Std}[\text {AVar}_{\phi _1}]$	${\mathbb {E}}[\text {AVar}_{\phi _2}]$	$\text {Std}[\text {AVar}_{\phi _2}]$
LD	1.967	0.9995	23.77	12.52
RM	1.328	0.6538	15.35	7.348
Irr	1.163	0.5698	14.84	7.738
RMIrr	0.8775	0.4228	10.68	5.306
GiIrr	$\mathbf {0.7148}$	0.3450	$\mathbf {8.798}$	4.490

**Fig. 10:**
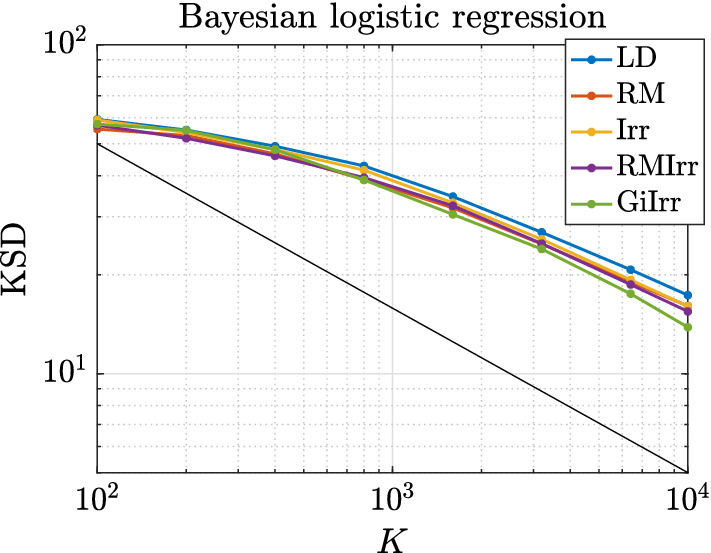
Kernelized Stein discrepancy plot for Bayesian logistic regression example. Black line has slope $-1/2$, which denotes the expected convergence rate

In Figure 10 we plot the KSD for the Bayesian logistic regression example. We see that GiIrr has slightly lower KSD than the other perturbations, but the differences are small. The theoretical slope of $K^{-1/2}$ is still realized.

### Independent component analysis

Our last example considers the problem of blind signal separation addressed in Welling and Teh (2011) and Amari et al. (1996). This problem yields a posterior that is strongly non-Gaussian and multi-modal, and we show that GiIrr has substantially better sampling performance over standard reversible and irreversible perturbations. Suppose there are *m* separate unknown independent signals $s^i(t)$ for $i = 1,\ldots , m$ that are mixed by mixing matrix $\mathbf {M}\in {\mathbb {R}}^{d\times d}$. Suppose we can observe the mixed signals $X(t) = \mathbf {M} s(t)$ for *N* instances in time. The goal of independent component analysis is to infer a de-mixing matrix $\mathbf {W}$ such that the *m* signals are recovered up to a nonzero constant and permutation. As such, this problem is generally ill-posed, but is suitable to be considered in a Bayesian context. The ICA literature states that, based on real-world data, it is best to assume a likelihood model with large kurtosis. Following (Welling and Teh 2011; Amari et al. 1996), let $p(y_i) = \frac{1}{4} \text {sech}^2\left( \frac{1}{2} y_i\right)$. The prior on the weights $\mathbf {W}_{ij}$ is Gaussian with zero mean and precision $\lambda$. The posterior is equal to

31 $$\begin{aligned} p(\mathbf {W}|X) \propto |\det \mathbf {W}| \prod _{i = 1}^m p(\mathbf {w}_i^T \mathbf {x}) \prod _{ij} {\mathcal {N}}(\mathbf {W}_{ij}; 0,\lambda ^{-1}). \end{aligned}$$

The gradient of the log posterior with respect to the matrix $\mathbf {W}$ is then

32 $$\begin{aligned} f(\mathbf {W})&= \nabla _\mathbf {W}\log p(\mathbf {W}|X) \nonumber \\&= \left( N(\mathbf {W}^T)^{-1} -\sum _{n = 1}^N \tanh \left( \frac{1}{2} \mathbf {y}_n \right) \mathbf {x}_n^T \right) - \lambda \mathbf {W}. \end{aligned}$$

It is suggested in Amari et al. (1996) that the natural gradient should be used instead of the gradient we see here above to account for the information geometry of the problem. Specifically, Teh et al. (2016), Amari et al. (1996) post-multiply the gradient by $\mathbf {W}^T\mathbf {W}$ and arrive at the so-called natural gradient of the system

33 $$\begin{aligned} {\mathcal {D}}_{\mathbf {W}} := \left( N\mathbf {I}_d - \sum _{n = 1}^N \tanh \left( \frac{1}{2} \mathbf {y}_n \right) \mathbf {y}_n^T \right) \mathbf {W}-\lambda \mathbf {W}\mathbf {W}^T\mathbf {W}. \end{aligned}$$

In the context of RMLD, this is equivalent to perturbing the system with a reversible perturbation with $\tilde{\mathbf {B}}(\mathbf {W}) =\mathbf {W}^T\mathbf {W}\otimes \mathbf {I}_d$ pre-multipled in front of the vectorized gradient. That is, we have

$$\begin{aligned} \text {vec}(f(\mathbf {W})\mathbf {W}^T\mathbf {W}) = (\mathbf {W}^T\mathbf {W} \otimes \mathbf {I}_d) \text {vec}f(\mathbf {W}). \end{aligned}$$

This choice of reversible perturbation, however, may not be sufficient for accelerating the convergence of Langevin dynamics as $\tilde{\mathbf {B}}(\mathbf {W})-\mathbf {I}_{d^2}$ is not positive definite throughout the state space (Rey-Bellet and Spiliopoulos 2016). Instead, we choose the reversible perturbation $\mathbf {B}(\mathbf {W}) = \mathbf {I}_{d^2} + (\mathbf {W}^\top \mathbf {W}\otimes \mathbf {I}_d) = ((\mathbf {I}_d + \mathbf {W}^\top \mathbf {W})\otimes \mathbf {I}_d).$

**Fig. 11:**
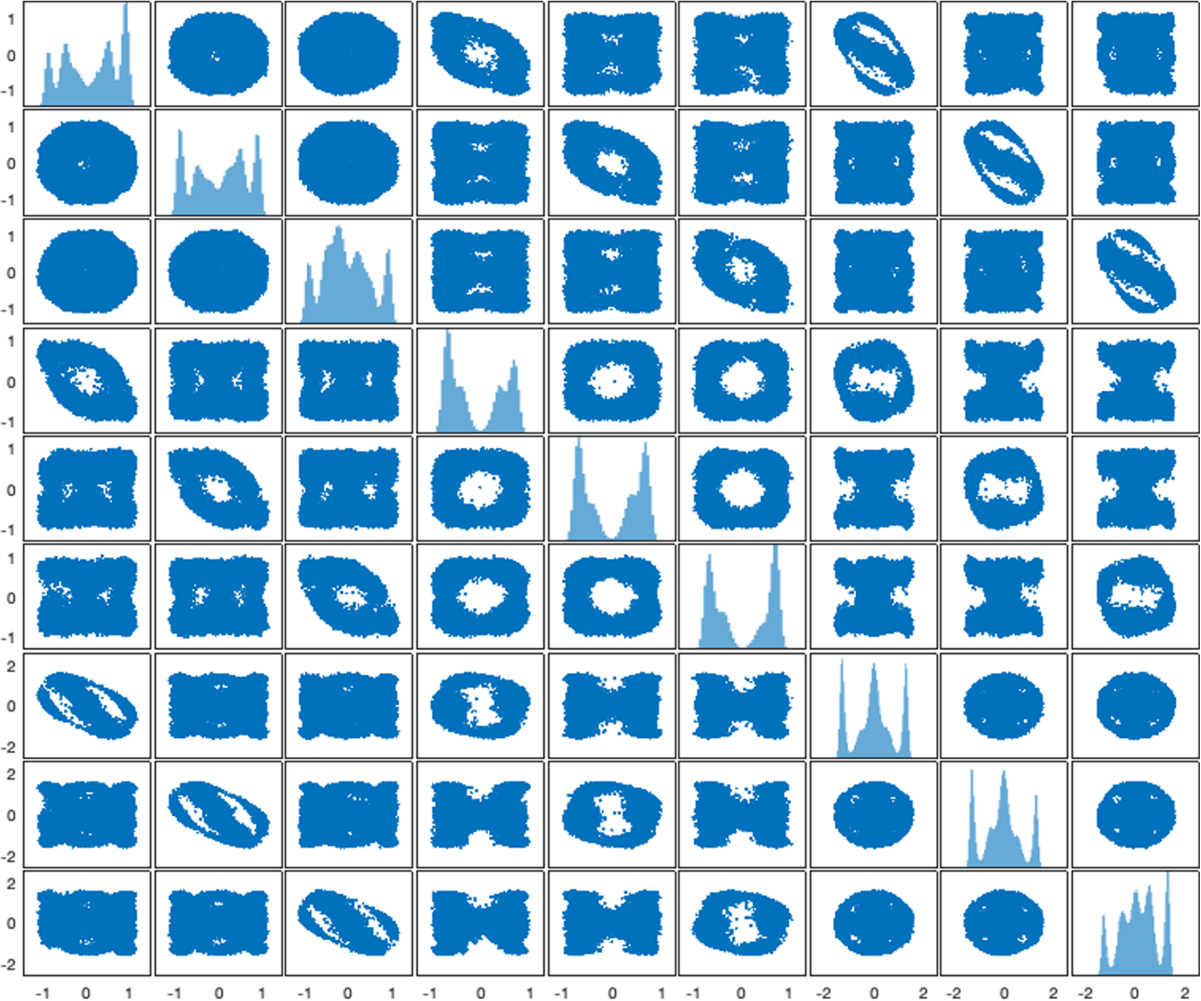
Posterior distribution sampled with standard Langevin with a deterministic gradient with $T = 10000$ and $h = 10^{-4}$. Notice that the system is very multimodal and non-Gaussian

**Fig. 12:**
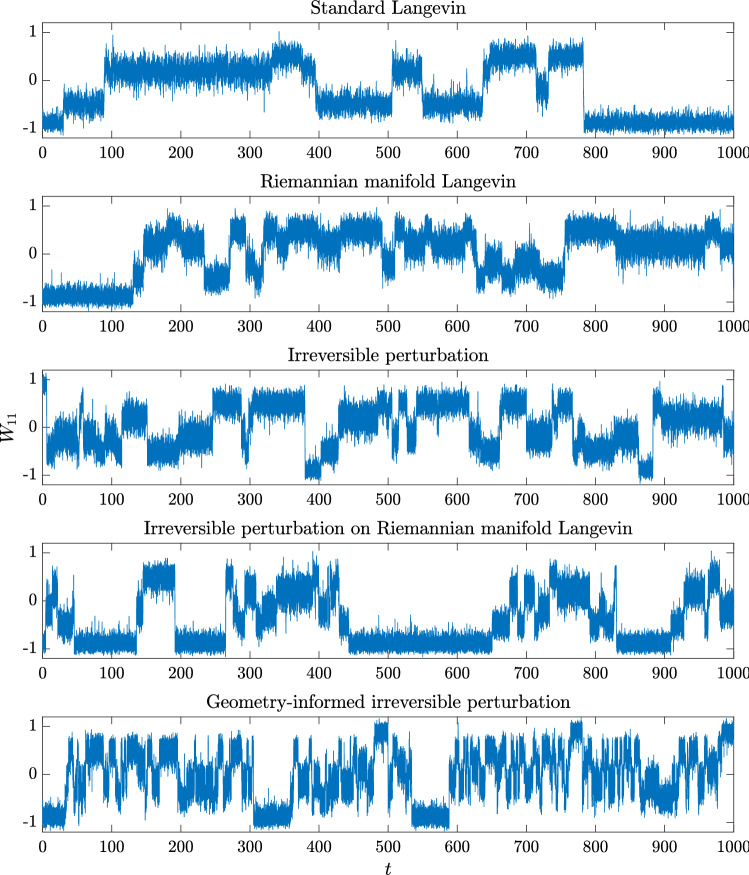
Trace plots of the $W_{11}$ marginal

We construct the GiIrr term as follows. To take advantage of the matrix structure of the reversible perturbation, we choose the skew-symmetric matrix such that it acts within the computation of the natural gradient. We choose $\mathbf {J} = (\mathbf {I}_d \otimes \mathbf {C}_0) + (\mathbf {C}_0 \otimes \mathbf {I}_d)$ where $\mathbf {C}_0$ has the same sign pattern as (22) but such that $\mathbf {J}$ has matrix norm equal to 1. Then the geometry-informed irreversible perturbation is

$$\begin{aligned} \frac{1}{2}\mathbf {B}(\mathbf {W})\mathbf {J} + \frac{1}{2}\mathbf {J} \mathbf {B}(\mathbf {W})&=((\mathbf {I}_d+\mathbf {W}^T\mathbf {W})\otimes \mathbf {C}_0)+(\mathbf {C}_0 \otimes \mathbf {I}_d)\\&\quad +\frac{1}{2}(\mathbf {W}^T\mathbf {W}\mathbf {C}_0 \otimes \mathbf {I}_d)\\&\quad +\frac{1}{2}(\mathbf {C}_0 \mathbf {W}^T\mathbf {W}\otimes \mathbf {I}_d). \end{aligned}$$

To simulate the RM and GiIrr systems, correction terms (such as $\nabla \cdot \mathbf {B}(\theta )$) need to be computed. The correction terms are derived using the symbolic algebra toolbox in MATLAB. Since the perturbations are vectors of polynomials, the symbolic algebra toolbox can easily derive and efficiently evaluate the correction terms.

For the numerical experiments, we synthetically generate $m = 3$ signals, one of which is Laplace distributed, and two are distributed according to the squared hyperbolic secant distribution. The posterior distribution is $d = 9$ dimensional, there are a total of $N = 400$ data points, and the gradient is approximated by subsampling $n = 40$ data points per estimate. The initial condition here is chosen to be a diagonal matrix with either $+1$ or $-1$ entries, which are chosen randomly. Since the posterior is nine-dimensional and highly multimodal, it is difficult to evaluate its marginal densities directly, i.e., without sampling. Instead, we establish a baseline reference density by simulating the standard Langevin dynamics with exact computation of the likelihood over all the data over $T = 10000$ with $h = 10^{-4}$. One- and two-dimensional marginals of this baseline posterior distribution are plotted in Figure 11. The two-dimensional marginals highlight the challenges of sampling from this posterior. In Figure 12, we plot trace plots of the $\mathbf {W}_{11}$ variable for each system. By visual inspection, we see that that mixing is best for the geometry-informed irreversibly perturbed system. One can intuitively expect that with better mixing, the geometry-informed irreversibility should yield better estimation performance than the other systems. We assess this quantitatively below.

**Fig. 13:**
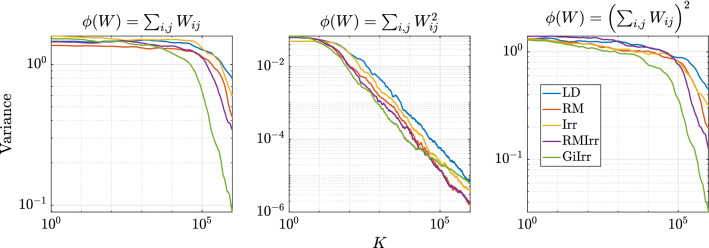
Variance of running average estimators. For the second moment (middle plot), there is less difference among the samplers as the distribution is quite symmetric, and one can properly estimate the second moment even if the samplers are stuck in a single mode. GiIrr is able to estimate the observable with cross-moments (right plot) better than the other samplers

As in the previous example, we simulate the five systems and compute the asymptotic variances of two observables for each system. Each system is simulated independently 100 times up to time $T = 2000$ with $h = 2\times 10^{-5}$. The smaller step size is to account for the additional stiffness irreversible perturbations introduce. Since the true mean of the posterior distribution is unknown, and because standard sampling methods fail to adequately sample from the posterior distribution to get a reasonable estimate for the mean, we only plot the variances of the selected observables with respect to *K* in Figure 13. To compute the asymptotic variance, we allot a burn-in time of $T_b = 20$. The observables we estimate are $\phi _1(\mathbf {W}) = \sum _{i,j} \mathbf {W}_{ij}$, $\phi _2(\mathbf {W}) = \sum _{i,j} \mathbf {W}_{ij}^2$, and $\phi _3(\mathbf {W}) = \left( \sum _{i,j}\mathbf {W}_{ij} \right) ^2$. The asymptotic variance numbers confirm that the faster mixing observed in the geometry-adapted irreversible perturbation does lead to a better sampling method. The values of the asymptotic variance are reported in Table 6. The results for the asymptotic variance and variance of $\phi _2$ are somewhat noisy, which is why GiIrr may appear to perform similarly as the other sampling methods. When estimating the posterior mean and an observable ($\phi _3$) that includes cross-moments, the geometry-informed irreversible perturbation outperforms standard irreversibility applied to the reversible perturbation.

**Table 6:** Asymptotic variance estimates for the ICA example

	${\mathbb {E}}[\text {AVar}_{\phi _1}]$	$\text {Std}[\text {AVar}_{\phi _1}]$	${\mathbb {E}}[\text {AVar}_{\phi _2}]$	$\text {Std}[\text {AVar}_{\phi _2}]$	${\mathbb {E}}[\text {AVar}_{\phi _3}]$	$\text {Std}[\text {AVar}_{\phi _3}]$
LD	80.40	23.61	$9.445 \times 10^{-4}$	$3.616\times 10^{-4}$	50.17	17.52
RM	53.01	12.22	$5.489\times 10^{-4}$	$1.607\times 10^{-4}$	26.75	8.442
Irr	52.38	17.27	$6.854\times 10^{-4}$	$2.035\times 10^{-4}$	27.02	9.134
RMIrr	39.20	10.36	$5.794\times 10^{-4}$	$1.873\times 10^{-4}$	19.47	6.086
GiIrr	$\mathbf {15.50}$	4.441	$1.253\times 10^{-3}$	$3.800\times 10^{-4}$	$\mathbf {6.381}$	1.777

**Fig. 14:**
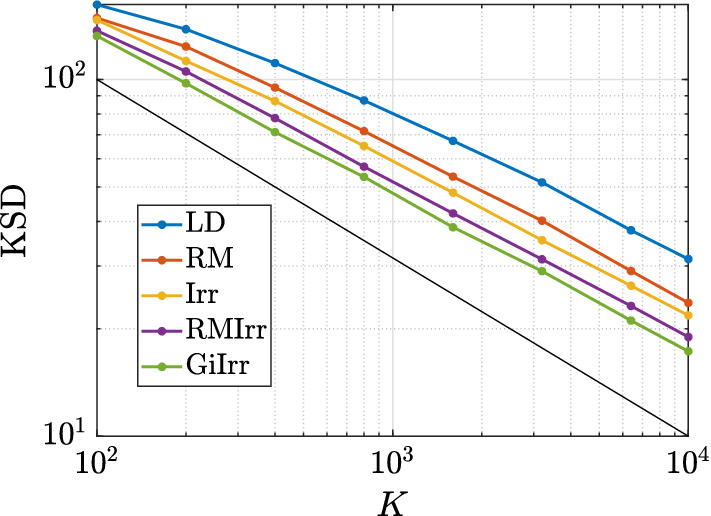
Kernelized Stein discrepancy plot for the ICA example. Black line has slope $-1/2$, which denotes the expected convergence rate

In Figure 14 we plot the convergence of KSD for the ICA example. GiIrr yields lower KSD than the other perturbations in this example, and the theoretical slope of $K^{-1/2}$ is also realized.

## Conclusion

We presented a novel irreversible perturbation, GiIrr, that accelerates the convergence of Langevin dynamics. By introducing an irreversible perturbation that incorporates any given underlying reversible perturbation, which can also be interpreted as defining a Riemannian metric, we have shown through numerical examples that geometry-informed irreversible perturbations outperform those that are not informed as such. In the examples, we found that GiIrr seems to perform best when the target distribution is highly non-Gaussian.

Most of our numerical examples used stochastic gradients to cut down on computational effort in sampling each trajectory. This demonstrates that SGLD can be used in conjunction with irreversibility for practical computations.

We also provided some analysis on how irreversibility interacts with discretization of the SDE systems. Irreversibility introduces additional stiffness into the system, which may lead to additional bias or variance in the estimator. For practical purposes, one can simply choose a small enough step size so that the asymptotic bias and variance are sufficiently small. At the same time, we note an example (see Appendix A) where the introduction of the irreversible term, once discretized, leads to no improvement in the long term average estimator.

Future work could study the use of novel integrators which circumvent stiffness. For example, Jianfeng and Spiliopoulos (2018) uses a multiscale integrator, but it is not readily adapted to the data-driven setting of Bayesian inference. Another direction for future work is to theoretically characterize the performance of the geometry-informed irreversible perturbation and to compare it with that of other perturbations. A starting point for such an analysis could be the general results of Rey-Bellet and Spiliopoulos (2016), in particular the large deviations Theorem 1 together with Propositions 2–4 therein. Preliminary investigation of this direction showed that it is a promising avenue for a theoretical investigation, but non-trivial work and a finer analysis are needed to demonstrate the effects of this class of irreversible perturbations. We leave this for future work, as such an analysis is outside the scope of this paper. Our goal in this paper has been to introduce the perturbation and showcase its potential through simulation examples.

## Figures and Tables

**Fig. 15:**
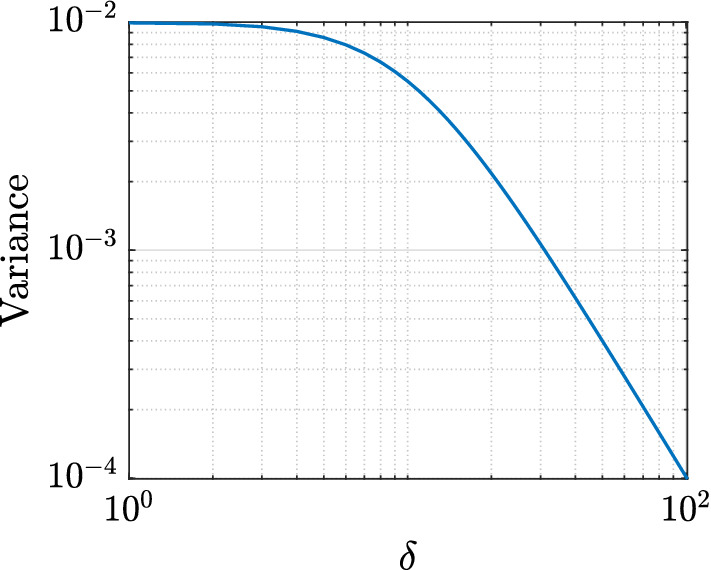
Variance for different $\delta$, fixed *h*

**Fig. 16:**
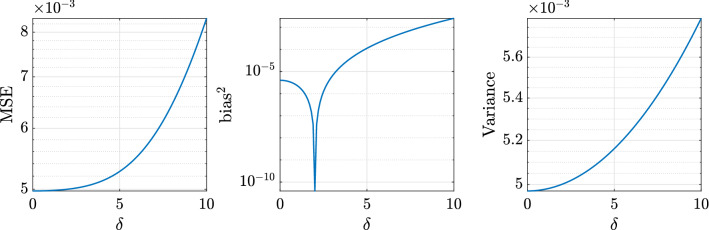
(Squared) bias and variance of $\phi (\theta ) = \Vert \theta \Vert ^2$ for varying levels of irreversibility

## Appendix A The effects of discretization

In this section, we study the effects of discretization in the setting of an irreversibly perturbed Langevin system. Results in full generality are, as yet, elusive; therefore we only consider a Gaussian example, as it still provides insight into how irreversibility interacts with discretization in impacting the asymptotic and finite sample bias and variance of the long term average estimator. While we do not present the results when a stochastic gradient is used, we note that the results are similar and can be easily extended based on what we present here. Recall that,

$$\begin{aligned} \mathbf {A}&= \frac{1}{2}(\mathbf {I}+\mathbf {J})({\varvec{\Gamma }}_\theta + N {\varvec{\Gamma }}_X),\\ \mathbf {D}&= \frac{1}{2}(\mathbf {I}+\mathbf {J}) \left( {\varvec{\Gamma }}_X \sum _{i = 1}^N X_{i} \right) , \text { where } \mathbf {J}= \delta \begin{bmatrix} 0 &{} 1 \\ -1 &{} 0 \end{bmatrix} \end{aligned}$$

For this analysis, all precision matrices are $2\times 2$ scalar matrices. That is, we assume ${\varvec{\Gamma }}_\theta =\sigma _\theta ^{-2}\mathbf {I}$, ${\varvec{\Gamma }}_X = \sigma _X^{-2}\mathbf {I}$. This is distinct from the example in Section 3.2, since the precision matrices there are diagonal but not scalar. Let $b = \frac{1}{2\sigma _X^2}$ and $S_X =\sum _{k = 1}^N X_i$, so that $\mathbf {D}= b(\mathbf {I}+\mathbf {J}) S_X$.

We summarize our findings here. For fixed discretization size *h* and scalar precision matrices as defined above, and introducing the irreversible perturbation scaled by $\delta$, we find the following:

- The asymptotic bias for linear observables is zero, that is, ${\mathbb {E}}[\theta _\infty ] = \mu _p$;
- The asymptotic variance for linear observables increases. We found that
  34 $$\begin{aligned} \text {Tr}\,\text {Var}[\theta _\infty ] = \frac{2}{2a-ha^2(1+\delta ^2)} \end{aligned}$$
  where $$a = 0.5 (1/\sigma _\theta ^2 + N/\sigma _X^2)$$;
- The finite time estimator for the observable $\phi (\theta ) =\theta _1 + \theta _2$ has lower bias and variance;
- The finite time estimator for the observable $\phi (\theta ) =\Vert \theta \Vert ^2$ has higher bias and variance.

We focus on the finite time results and omit the asymptotic results, since the the former case is of more practical interest. The computations related to both are similar.

**Finite time analysis: bias for linear observables.** We study how the magnitude of the irreversibility, characterized by $\delta$, impacts the mean-squared error $\text {MSE} = {\mathbb {E}}\left[ \Vert \bar{\theta }_K-\mu _p\Vert ^2\right]$ where $\bar{\theta }_K = \frac{1}{K}\sum _{k = 0}^{K-1} \theta _k$. We approach this quantity via its bias-variance decomposition:

35 $$\begin{aligned} \text {MSE} = \left\| {\mathbb {E}}[\bar{\theta }_{K}] - \mu _p \right\| ^2 + \text {Tr}\,\text {Var}\left( \bar{\theta }_K\right) . \end{aligned}$$

First, we compute the expected value of the sample average ${\mathbb {E}}\left[ \bar{\theta }_K\right] = \frac{1}{K} \sum _{k = 0}^{K-1} {\mathbb {E}}[\theta _k].$ For simplicity, we assume that the initial condition is always $\theta _0 = \mathbf {0}$. For any *k*, we have

$$\begin{aligned} {\mathbb {E}}[\theta _k]&= (\mathbf {I}-h\mathbf {A}){\mathbb {E}}[\theta _{k-1}] + h\mathbf {D}\\&= (\mathbf {I}-h\mathbf {A})^k \theta _0 + h\sum _{n = 0}^{k-1} (\mathbf {I}-h\mathbf {A})^n \mathbf {D}\\&= h(\mathbf {A}h)^{-1}(\mathbf {I}-(\mathbf {I}-h\mathbf {A})^{k})\mathbf {D}\\&= \mathbf {A}^{-1}\mathbf {D}- \mathbf {A}^{-1}(I-h\mathbf {A})^k \mathbf {D}. \end{aligned}$$

This yields

$$\begin{aligned} {\mathbb {E}}[\bar{\theta }_K]&= \frac{1}{K}\sum _{k = 0}^{K-1} \left( \mathbf {A}^{-1}\mathbf {D}- \mathbf {A}^{-1}(\mathbf {I}-h\mathbf {A})^k \mathbf {D}\right) \\&= \mathbf {A}^{-1}\mathbf {D}- \frac{1}{K}\mathbf {A}^{-1}(\mathbf {A}h)^{-1}(\mathbf {I}-(\mathbf {I}-h\mathbf {A})^K)\mathbf {D}. \end{aligned}$$

Since $\mu _p = \mathbf {A}^{-1}\mathbf {D}$, the bias is

$$\begin{aligned} \text {bias} = -\frac{1}{Kh}\mathbf {A}^{-2}(\mathbf {I}-(\mathbf {I}-h\mathbf {A})^K)\mathbf {D}. \end{aligned}$$

The norm of the bias can in fact be computed. Note that $\mathbf {A}^2=(1+\delta ^2)a^2\mathbf {I}$ and we have

$$\begin{aligned}&\Vert \text {bias}\Vert ^2 \\&\quad = \frac{1}{K^2h^2} \mathbf {D}^T (\mathbf {I}-(\mathbf {I}-h\mathbf {A}^T)^K)\mathbf {A}^{-2T}\mathbf {A}^{-2} (\mathbf {I}-(\mathbf {I}-h\mathbf {A})^K) \mathbf {D}\\&\quad = \frac{1}{K^2h^2a^4(1+\delta ^2)^2}\mathbf {D}^T(\mathbf {I}-(\mathbf {I}-h\mathbf {A}^T)^K)(\mathbf {I}-(\mathbf {I}-h\mathbf {A})^K) \mathbf {D}\\&\quad = \frac{b^2}{K^2h^2a^4(1+\delta ^2)^2}S_X^T\\&\qquad (\mathbf {I}+\mathbf {J})^T(\mathbf {I}-(\mathbf {I}-\mathbf {A}^Th)^K) (\mathbf {I}-(\mathbf {I}-h\mathbf {A})^K) (\mathbf {I}+\mathbf {J}) S_X. \end{aligned}$$

The inner matrix can be computed. Since each matrix above is simultaneously diagonalizable, we only need to consider the eigenvalues of each of the above matrices. Note that $\mathbf {I}+\mathbf {J}$ is a normal matrix, so we may write the eigenvalue decomposition $\mathbf {I}+\mathbf {J}=\mathbf {PDP}^*$, where $^*$ denotes conjugate transpose, $\mathbf {Q} = \text {diag}(1+i\delta ,1-i\delta )$, and

$$\begin{aligned} \mathbf {P} = \frac{1}{\sqrt{2}}\begin{bmatrix} 1 &{} 1 \\ i &{} -i \end{bmatrix} \end{aligned}$$

is orthogonal. Now note that

$$\begin{aligned}&\mathbf {I}-(\mathbf {I}-h\mathbf {A})^K \\&\quad = \mathbf {P} \begin{bmatrix} 1-(1-ah(1+i\delta ))^K &{} 0 \\ 0 &{} 1-(1-ah(1-i\delta ))^K \end{bmatrix} \mathbf {P}^*, \end{aligned}$$

which implies

$$\begin{aligned} (\mathbf {I}-(\mathbf {I}-h\mathbf {A}^T)^K)(\mathbf {I}-(\mathbf {I}-h\mathbf {A})^K)= |1-(1-ah(1+i\delta )^K)|^2\mathbf {I}. \end{aligned}$$

Using the fact that $(\mathbf {I}+\mathbf {J})^T(\mathbf {I}+\mathbf {J}) = (1+\delta ^2)I$, and we have the following

36 $$\begin{aligned} \Vert \text {bias}\Vert ^2 = \frac{b^2}{K^2h^2a^4(1+\delta ^2)} |1 -(1-a(1+i\delta )h)^K|^2 \Vert S_X\Vert . \end{aligned}$$

To simplify further, we write $1-a(1+i\delta )h = re^{i\theta }$ where $r^2 = (1-ah)^2 + \delta ^2a^2h^2$, and $\tan \theta = \delta a h/ (1-ah)$. Then we obtain

$$\begin{aligned} \Vert \text {bias}\Vert ^2&= \frac{b^2}{K^2h^2a^4(1+\delta ^2)} |1 -r^Ke^{i\theta K}|^2 \Vert S_X \Vert ^2 \\&= \frac{b^2}{K^2h^2a^4(1+\delta ^2)} (1+r^{2K}-2r^K \cos K\theta )\Vert S_X \Vert ^2. \end{aligned}$$

We know that $r<1$, since otherwise, the numerical scheme would be unstable. It is easy to see that for large, but not infinite, *K*, the bias decays as ${\mathcal {O}}(1/(Kh\sqrt{1+\delta ^2}))$, so the introduction of irreversibility decreases the constant in front of the expression and therefore slightly improves the convergence of the bias.

**Finite time analysis: variance for linear observables.** For simplicity, we assume $\theta _0 = 0$. We compute $\text {TrVar}(\bar{\theta }_K)$. We begin with

$$\begin{aligned} \text {Tr}\,\text {Var}(\bar{\theta }_K) =\text {Tr}\,{\mathbb {E}}[\bar{\theta }_K\bar{\theta }_K^T] -\text {Tr}\,{\mathbb {E}}[\bar{\theta }_K]{\mathbb {E}}[\bar{\theta }_K]^T \end{aligned}$$

and compute these terms separately. It is difficult to surmise a relationship between $\delta$ and $\text {Tr}\,\text {Var}(\bar{\theta }_K)$ even with exact formulas, so we appeal to plots of the expressions to see that the variance decreases with irreversibility. We computed ${\mathbb {E}}[\bar{\theta }_K]$ in the previous section.

With the observation that

$$\begin{aligned} \mathbf {A}^{-2}(\mathbf {I}-(\mathbf {I}-h\mathbf {A})^K) {\mathbb {E}}[\mathbf {D}] = \frac{b}{a^2}\mathbf {PQ'P}^*{\mathbb {E}}[S_X] \end{aligned}$$

where

$$\begin{aligned} \mathbf {Q}' = \begin{bmatrix} \frac{1-(1-ah(1+i\delta ))^K}{1+i\delta } &{} 0 \\ 0 &{} \frac{1-(1-ah(1-i\delta ))^K}{1-i\delta }, \end{bmatrix} \end{aligned}$$

and $\mathbf {P}$ is defined in the previous section. We compute that

37 $$\begin{aligned} \text {Tr}\,{\mathbb {E}}[\bar{\theta }_K] {\mathbb {E}}[\bar{\theta }_K]^T&= \Vert \mu _p\Vert ^2 + \Vert \text {bias}\Vert ^2 - \frac{2b^2}{Kha^3(1+\delta ^2)}\nonumber \\&\quad \text {Re}\{(1-i\delta )(1-ah(1+i\delta ))^K \} \Vert {\mathbb {E}}[S_X]\Vert ^2. \end{aligned}$$

The other term is more complicated and needs to be approached more gingerly. Observe that

$$\begin{aligned} \text {Tr}\,{\mathbb {E}}[\bar{\theta }_K\bar{\theta }_K^T ]&= \frac{1}{K^2} \sum _{i,j = 1}^K \text {Tr}\,{\mathbb {E}}[\theta _i\theta _j^T] = \frac{1}{K^2}\\&\quad \left( \sum _{i = 0}^{K-1} \text {Tr}\,{\mathbb {E}}[\theta _i\theta _i^T] + 2\sum _{i<j = 0}^{K-1} \text {Tr}\,{\mathbb {E}}[\theta _i\theta _j^T] \right) . \end{aligned}$$

We take each term individually. To compute ${\mathbb {E}}[\theta _k\theta _k^T]$, it is actually better to consider the covariance matrix of $\theta _k$, $\Sigma _{k} = {\mathbb {E}}[\theta _k\theta _k^T] - {\mathbb {E}}[\theta _k]{\mathbb {E}}[\theta _k]^T$.

We first compute

$$\begin{aligned} {\mathbb {E}}[ \theta _{k} \theta _{k}^T ]&= (\mathbf {I}-h\mathbf {A}) {\mathbb {E}}[\theta _{k-1} \theta _{k-1}^T] (\mathbf {I}-h\mathbf {A})^T + h^2 \mathbf {D}\mathbf {D}^T + h \mathbf {I}\\&\quad + (\mathbf {I}-h\mathbf {A}) {\mathbb {E}}[\theta _{k-1}] \mathbf {D}^T h + h\mathbf {D}{\mathbb {E}}[ \theta _{k-1}^T](\mathbf {I}-h\mathbf {A})^T \\ {\mathbb {E}}[\theta _{k}]{\mathbb {E}}[\theta _{k}]^T&= (\mathbf {I}-h\mathbf {A}) {\mathbb {E}}[\theta _{k-1}]{\mathbb {E}}[\theta _{k-1}]^T (\mathbf {I}-h\mathbf {A})^T \\&\quad + \mathbf {D}{\mathbb {E}}[\theta _{k-1}]^T (\mathbf {I}-h\mathbf {A})^T \\&\quad + (\mathbf {I}-h\mathbf {A}) {\mathbb {E}}[\theta _{k-1}]\mathbf {D}+ h^2\mathbf {D}\mathbf {D}^T \end{aligned}$$

which imply the following recurrence relation. Assuming $\Sigma _0 = \mathbf {0}$, we have

$$\begin{aligned}&\Sigma _{k} = (\mathbf {I}-h\mathbf {A}) \Sigma _{k-1}(\mathbf {I}-h\mathbf {A})^T + h\mathbf {I}\\&\quad = h \sum _{n = 0}^{k-1}\left( (\mathbf {I}-h\mathbf {A})(\mathbf {I}-h\mathbf {A})^{T}\right) ^n \\&\quad = h\sum _{n = 0}^{k-1}(\mathbf {I}-(\mathbf {A}+\mathbf {A}^T)h + h^2\mathbf {A}\mathbf {A}^T)^n \\&\quad = ((\mathbf {A}+\mathbf {A}^T)-h^2\mathbf {A}\mathbf {A}^T)^{-1}(\mathbf {I}-(\mathbf {I}-(\mathbf {A}+\mathbf {A}^T)h+\mathbf {A}\mathbf {A}^T h^2)^k). \end{aligned}$$

Let $s = 1-2ah + h^2a^2(1+\delta ^2)$, then, by recalling that $\mathbf {A}+\mathbf {A}^T = 2a\mathbf {I}$ and $\mathbf {A}\mathbf {A}^T = a^2(1+\delta ^2)\mathbf {I}$, the above sum is equal to $\frac{1-s^k}{1-s} h\mathbf {I}$. Therefore,

38 $$\begin{aligned} \text {Tr}\,\Sigma _k = \frac{2h(1-s^k)}{1-s}. \end{aligned}$$

Meanwhile note that

$$\begin{aligned} {\mathbb {E}}[\theta _k]&= \mu _p -\mathbf {A}^{-1} (\mathbf {I}-h\mathbf {A})^k \mathbf {D}. \end{aligned}$$

Therefore,

$$\begin{aligned}&\text {Tr}\,{\mathbb {E}}[\theta _k]{\mathbb {E}}[\theta _k]^T = {\mathbb {E}}[\theta _k]^T{\mathbb {E}}[\theta _k]\\&\quad = \Vert \mu _p\Vert ^2 + \mathbf {D}^T(\mathbf {I}-h\mathbf {A}^T)^k \mathbf {A}^{-T}\mathbf {A}^{-1}\\&\qquad (\mathbf {I}-h\mathbf {A})^k\mathbf {D}-2\mu _p^T\mathbf {A}^{-1}(\mathbf {I}-h\mathbf {A})^k \mathbf {D}\\&\quad = \Vert \mu _p\Vert ^2 + \frac{s^k b^2}{a^2(1+\delta ^2)} \Vert S_X\Vert ^2-2\mu _p^T\mathbf {A}^{-1}(\mathbf {I}-h\mathbf {A})^k \mathbf {D}. \end{aligned}$$

We now take the sum for each expression from $k = 0$ to $K-1$. We have

$$\begin{aligned} \sum _{i =0}^{K-1} \text {Tr}\,\Sigma _i&= 2h\left( \frac{K}{1-s} -\sum _{i = 0}^{K-1}\frac{s^i}{1-s}\right) \\&= 2h\left( \frac{K}{1-s}- \frac{1-s^K}{(1-s)^2}\right) \end{aligned}$$

and

$$\begin{aligned} \sum _{i = 0}^{K-1}{\mathbb {E}}[\theta _i]^T{\mathbb {E}}[\theta _i]&= K\Vert \mu _p\Vert ^2 + \frac{(1-s^K) b^2}{a^2(1+\delta ^2)(1-s)}\\&\quad \Vert S_X\Vert ^2-2\mu _p^T\mathbf {A}^{-1}(h\mathbf {A})^{-1}(\mathbf {I}-(\mathbf {I}-h\mathbf {A})^K) \mathbf {D}\\&= K\Vert \mu _p\Vert ^2 + \frac{(1-s^K) b^2}{a^2(1+\delta ^2)(1-s)}\\&\quad \Vert S_X\Vert ^2-2\mu _p^Th^{-1}\mathbf {A}^{-2}(\mathbf {I}-(\mathbf {I}-h\mathbf {A})^K) \mathbf {D}. \end{aligned}$$

For the cross-terms, observe that we may write

39 $$\begin{aligned} \sum _{i<j = 0}^{K-1} \theta _i\theta _j^T = \sum _{i = 0}^{K-1} \theta _i \sum _{j = i+1}^{K-1} \theta _j^T \end{aligned}$$

which can be simplified further. First note that

$$\begin{aligned} \theta _j&= (\mathbf {I}-h\mathbf {A}) \theta _{j-1} + \mathbf {D}h + \sqrt{h}\xi _{j-1} \\&= (\mathbf {I}-h\mathbf {A})^{j-i} \theta _i + h\sum _{n = 0}^{j-1-i} (\mathbf {I}-h\mathbf {A})^{n} \mathbf {D}\\&\quad + \sqrt{h} \sum _{n = 0}^{j-i-1} (\mathbf {I}-h\mathbf {A})^{n} \xi _{j-1-n}. \end{aligned}$$

Plugging this expression into the double sum above, we have

40 $$\begin{aligned}&\sum _{i =0}^{K-1} \theta _i \sum _{j = i+1}^{K-1} \left[ \theta _i^T(\mathbf {I}-\mathbf {A}^Th)^{j-i} + h \sum _{n = 0}^{j-1-i} \mathbf {D}^T(\mathbf {I}-\mathbf {A}^Th)^n \right. \nonumber \\&\qquad \quad \left. + \sqrt{h}\sum _{n = 0}^{j-i-1} \xi _{j-1-n}^T(\mathbf {I}-\mathbf {A}^Th)^n \right] . \end{aligned}$$

Taking expectations, we have

$$\begin{aligned}&\sum _{i = 0}^{K-1} \sum _{j = i+1}^{K-1} \left[ {\mathbb {E}}[\theta _i\theta _i^T](\mathbf {I}-h\mathbf {A}^T)^{j-i} + h{\mathbb {E}}[\theta _i]\mathbf {D}^T \sum _{n = 0}^{j-1-i} (\mathbf {I}-h\mathbf {A}^T)^n \right] \\&\quad = \sum _{i = 0}^{K-1} \sum _{j = i+1}^{K-1} \left[ {\mathbb {E}}[\theta _i\theta _i^T](\mathbf {I}-h\mathbf {A}^T)^{j-i}\right. \\&\qquad \quad \left. +h{\mathbb {E}}[\theta _i]\mathbf {D}^T (\mathbf {A}^Th)^{-1}(\mathbf {I}-(\mathbf {I}-\mathbf {A}^Th)^{j-i}) \right] . \end{aligned}$$

Carrying out the computation for the first term, we have

$$\begin{aligned} F&= \sum _{i = 0}^{K-1} {\mathbb {E}}[\theta _i\theta _i^T] \sum _{j = i+1}^{K-1} (\mathbf {I}-\mathbf {A}^Th)^{j-i} \\&= \sum _{i = 0}^{K-1} {\mathbb {E}}[\theta _i\theta _i^T] (\mathbf {I}-\mathbf {A}^Th)(\mathbf {A}^Th)^{-1}(\mathbf {I}-(\mathbf {I}-\mathbf {A}^Th)^{K-1-i}). \end{aligned}$$

For the second term we have,

$$\begin{aligned}&\sum _{i = 0}^{K-1} {\mathbb {E}}[\theta _i]\mu _p^T \sum _{j = i+1}^{K-1} (\mathbf {I}-(\mathbf {I}-\mathbf {A}^Th)^{j-i})\\&\quad = \sum _{i = 0}^{K-1} {\mathbb {E}}[\theta _i] \mu _p^T \left[ (K-1-i)\mathbf {I}- (\mathbf {I}-\mathbf {A}^Th)(\mathbf {A}^Th)^{-1} \right. \\&\qquad \quad \left. (\mathbf {I}-(\mathbf {I}-\mathbf {A}^Th)^{K-1-i}) \right] . \end{aligned}$$

The summations are difficult to compute precisely, so we compute them by direct evaluation instead. For simplicity, we assume that $\mu _p = {\mathbb {E}}[S_X] = [0,0]^T$, $\sigma _X$ and $\sigma _\theta$ are chosen such that $a=1$. For this scenario, the bias is zero and only the variance contributes to the MSE. The variance is

$$\begin{aligned} \text {Tr}\,[\text {Var}\overline{\theta }_K] = \frac{1}{K^2} \left( 2h\left( \frac{K}{1-s} - \frac{1-s^K}{(1-s)^2} \right) + 2\text {Tr}\,F\right) \end{aligned}$$

where $${\mathbb {E}}[\theta _i\theta _i^T] = \Sigma _i = \frac{1-s^i}{1-s}h\mathbf {I}.$$

In Figure 15 we plot the variance for varying choices of $\delta$. In this plots, $h = 0.001$, $K = 2\times 10^5$, and $\delta$ varies between zero and ten. We can clearly see that strengthening the irreversible perturbation leads to improvement of the squared bias and variance of the long term average estimator.

**Finite sample analysis for the quadratic observable** $\phi (\theta ) = \Vert \theta \Vert ^2$. The previous finite sample results for the observable $\phi (\theta ) = \theta _1 + \theta _2$ suggests that both the bias and variance of the long term average estimator goes down with a larger irreversible term. In this section, we show that this is actually a special case, and that when the observable is not linear, then the bias and variance may increase. We analyze the bias and variance of the long term average estimator of the observable $\phi (\theta ) = \Vert \theta \Vert ^2$. Define

41 $$\begin{aligned} \overline{\phi } = \int \phi (\theta ) \pi (\theta ) \text {d}\theta , \;\;\;\; \overline{\phi }_K = \frac{1}{K}\sum _{k = 0}^{K-1} \phi (\theta _k). \end{aligned}$$

As before, we assume that $\mu _p = [0,0]^T$, ${\mathbb {E}}[S_X] = \mathbf {0}$, and $\sigma _x$ and $\sigma _\theta$ are chosen such that $a=1$. We compute $|{\mathbb {E}}\overline{\phi }_K - \overline{\phi }|^2$ and $\text {Var}\overline{\phi }_k$ and see how they vary with $\delta$. From previous computations, we can show that

42 $$\begin{aligned} {\mathbb {E}}\overline{\phi }_K = 2h \left( \frac{1}{1-s} - \frac{1-s^K}{K(1-s)^2} \right) , \end{aligned}$$

where $s = 1-2ah + a^2h^2(1+\delta ^2)$. Given this, the only term left to compute is the variance of the second moment of this observable:

43 $$\begin{aligned} {\mathbb {E}}\left[ \left( \overline{\phi }_K\right) ^2 \right]&= \frac{1}{K^2} {\mathbb {E}}\left[ \left( \sum _{k = 0}^{K-1} \theta _k^T\theta _k \right) ^2 \right] \nonumber \\&= \frac{1}{K^2}\sum _{k = 0}^{K-1} {\mathbb {E}}\left[ (\theta _k^T\theta _k)^2 \right] \nonumber \\&\quad + \frac{2}{K^2}{\mathbb {E}}\sum _{k = 0}^{K-1} \sum _{l = k+1}^{K-1} (\theta _k^T\theta _k) (\theta _l^T \theta _l). \end{aligned}$$

To compute the first sum, consider the following:

$$\begin{aligned} \theta _k^T\theta _k&= \theta _{k-1}^T(\mathbf {I}-h\mathbf {A})^T(\mathbf {I}-h\mathbf {A}) \theta _{k-1} + h\xi _{k-1}^T\xi _{k-1} \\&\quad + 2\sqrt{h}\theta _{k-1}^T(\mathbf {I}-h\mathbf {A})^T \xi _{k-1} \end{aligned}$$

and so we have

$$\begin{aligned} (\theta _k^T \theta _k)^2&= s^2(\theta _{k-1}^T\theta _{k-1})^2 + h^2 (\xi _{k-1}^T \xi _{k-1})^2 \\&\quad + 4h (\xi _{k-1}^T(\mathbf {I}-h\mathbf {A}) \theta _{k-1})^2+2s\theta _{k-1}^T\theta _{k-1} h \xi _{k-1}^T \xi _{k-1} \\&\quad + 4 s\sqrt{h}(\theta _{k-1}^T\theta _{k-1}) \theta _{k-1}^T (\mathbf {I}-h\mathbf {A})^T\xi _{k-1}\\&\quad +4h^{3/2}(\xi _{k-1}^T\xi _{k-1}) \theta _{k-1}^T(\mathbf {I}-h\mathbf {A})^T\xi _{k-1}. \end{aligned}$$

Taking the expectation, we have

$$\begin{aligned} {\mathbb {E}}[(\theta _k^T\theta _k)^2]&= s^2 {\mathbb {E}}[(\theta _{k-1}^T \theta _{k-1})^2] + h^2 {\mathbb {E}}[(\xi _{k-1}^T\xi _{k-1})^2]\\&\quad +4h{\mathbb {E}}\left[ (\xi _{k-1}^T(\mathbf {I}-h\mathbf {A})\theta _{k-1})^2 \right] \\&\quad +2sh{\mathbb {E}}\left[ (\theta _{k-1}^T\theta _{k-1})(\xi _{k-1}^T\xi _{k-1}) \right] . \end{aligned}$$

After simplifying, we arrive at the following recurrence relation:

44 $$\begin{aligned} {\mathbb {E}}\left[ (\theta _k^T\theta _k)^2 \right] = s^2 {\mathbb {E}}\left[ (\theta _{k-1}^T\theta _{k-1})^2\right] + 8h^2 + 8sh {\mathbb {E}}\left[ \theta _{k-1}^T\theta _{k-1}\right] . \end{aligned}$$

Let $\beta _k = {\mathbb {E}}[(\theta _k^T\theta _k)^2]$, $\zeta _k = 8sh{\mathbb {E}}[\theta _k^T\theta _k]$, and $\kappa = 8h^2$. We have the following recurrence, which we solve

$$\begin{aligned} \beta _k&= s^2 \beta _{k-1} + \zeta _{k-1} + \kappa \\&= s^{2k} \beta _0 + \sum _{n = 0}^{k-1} s^{2n} \zeta _{k-n-1} + \sum _{n = 0}^{k-1} s^{2n} \kappa . \end{aligned}$$

From previous for the term $\zeta _k$, we have

$$\begin{aligned} \beta _k&= \sum _{n = 0}^{k-1} s^{2n} 8sh \cdot 2h \frac{1-s^{k-n-1}}{1-s} + \kappa \frac{1-s^{2k}}{1-s^2} \\&= \frac{16sh^2}{1-s} \sum _{n = 0}^{k-1} (s^{2n} -s^{k+n-1}) + \frac{8h^2(1-s^{2k})}{1-s^2} \\&= \frac{16sh^2}{1-s} \left( \frac{1-s^{2k}}{1-s^2} -s^{k-1} \frac{1-s^k}{1-s} \right) + \frac{8h^2(1-s^{2k})}{1-s^2}. \end{aligned}$$

Next we compute the summation of the cross terms. Define $R_k$ such that

45 $$\begin{aligned} \sum _{k = 0}^{K-1} R_k = \sum _{k = 0}^{K-1} \sum _{l = k+1}^{K-1} {\mathbb {E}}\left[ (\theta _k^T\theta _k)(\theta _l^T\theta _l)\right] . \end{aligned}$$

We write

$$\begin{aligned} \theta _l^T \theta _l&= s\theta _{l-1}^T\theta _{l-1} +h \xi _{l-1}^T\xi _{l-1} + 2\sqrt{h} \xi _{l-1}^T (\mathbf {I}-h\mathbf {A}) \theta _{l-1} \\&= s^{l-k} \theta _k^T\theta _k \\&\quad +\sum _{n = 0}^{l-k-1} hs^n \xi _{l-n-1}^T \xi _{l-n-1} + 2\sqrt{h} s^n \xi _{l-n-1}^T(\mathbf {I}-h\mathbf {A}) \theta _{l-n-1}. \end{aligned}$$

This implies that

$$\begin{aligned} R_k&= \sum _{l = k+1}^{K-1} \left( s^{l-k}{\mathbb {E}}[ (\theta _k^T\theta _k)^2] +\sum _{n = 0}^{l-k-1} 2h s^n {\mathbb {E}}[\theta _k^T\theta _k] \right) \\&= \sum _{l = k+1}^{K-1} \beta _ks^{l-k} + 2h{\mathbb {E}}[\theta _k^T\theta _k] \sum _{n = 0}^{l-k-1} s^n \\&= \sum _{l = k+1}^{K-1} \beta _k s^{l-k} + 2h{\mathbb {E}}[\theta _k^T\theta _k] \frac{1-s^{l-k}}{1-s} \\&= \beta _k \sum _{l = k+1}^{K-1} s^{l-k} +\frac{2h{\mathbb {E}}[\theta _k^T\theta _k]}{1-s} \sum _{l = k+1}^{K-1} 1-s^{l-k} \\&= \beta _k \frac{s-s^{K-k}}{1-s} + \frac{2h{\mathbb {E}}[\theta _k^T\theta _k]}{1-s} \left( K-1-k - \frac{s-s^{K-k}}{1-s} \right) . \end{aligned}$$

To summarize, we have

46 $$\begin{aligned} {\mathbb {E}}\left[ \left( \overline{\phi }_K \right) ^2 \right] =\frac{1}{K^2} \sum _{k = 0}^{K-1}\left( \beta _k +2 R_k \right) , \end{aligned}$$

the squared bias is $({\mathbb {E}}\overline{\phi }_K - 1)^2$ and the variance is ${\mathbb {E}}[(\overline{\phi }_K)^2] - {\mathbb {E}}[\overline{\phi }_K]^2$. These expressions are not simplifiable easily, so we plot these expressions and study their trends. In Figure 16, we plot the squared bias and variance for fixed *h* and *K* and varying $\delta$. In these plots, $h = 0.001$, $K = 2\times 10^5$, and $\delta$ varies between zero and ten. Notice that for these choices, both the squared bias and variance increases as $\delta$ grows, showing that for irreversibility provides no benefit, and in fact, harms the performance of the standard estimator.

### Remark A.1

When discretization is considered, sampling properties in this simple example in which the drift is proportional to the state cannot be improved upon using irreversibility. Let us now explain this phenomenon from a theoretical point of view. In Rey-Bellet and Spiliopoulos (2016), the authors show that adding an irreversible perturbation to the generator of the diffusion process may decrease the spectral gap, and will never increase it. They further prove that in the continuous case, decreasing the spectral gap then decreases the asymptotic variance. However this improvement is not strict, that is, irreversibility is only guaranteed to not increase the spectral gap.

Meanwhile, in Lelievre et al. (2013), the authors consider irreversibility only in the context of linear systems, and rigorously study optimal irreversible perturbations that accelerate convergence to the invariant distribution. Their results show that when the drift matrix is proportional to the identity matrix, the spectral gap cannot be widened. Proposition 4 in Lelievre et al. (2013) shows that the leading nonzero eigenvalue of the irreversibly perturbed drift matrix is bounded above by the leading nonzero eigenvalue of the original drift matrix and below by the trace of the original drift matrix over the dimension of the state space. The lower bound is then the optimal spectral gap. For a drift matrix that is a multiple of the identity, the upper and lower bounds are the same, which implies that the spectral gap can never decrease from its original value in the continuous case. After factoring in discretization, the irreversible perturbation increases stiffness of the system, which contributes to increased bias and variance in the resulting estimator.

## Declarations

### Code availability

Code can be made availble upon request.
